# Catabolism of β-5 linked aromatics by *Novosphingobium aromaticivorans*

**DOI:** 10.1128/mbio.01718-24

**Published:** 2024-07-16

**Authors:** Fletcher Metz, Abigail M. Olsen, Fachuang Lu, Kevin S. Myers, Marco N. Allemann, Joshua K. Michener, Daniel R. Noguera, Timothy J. Donohue

**Affiliations:** 1 DOE Great Lakes Bioenergy Research Center, University of Wisconsin, Madison, Wisconsin, USA; 2 Wisconsin Energy Institute, University of Wisconsin, Madison, Wisconsin, USA; 3 Laboratory of Genetics, University of Wisconsin, Madison, Wisconsin, USA; 4 Biosciences Division, Oak Ridge National Laboratory, Oak Ridge, Tennessee, USA; 5 Department of Civil and Environmental Engineering, University of Wisconsin, Madison, Wisconsin, USA; 6 Department of Bacteriology, University of Wisconsin, Madison, Wisconsin, USA; University of Washington School of Medicine, Seattle, Washington, USA

**Keywords:** enzymes, ligninolysis, genomics, biotechnology, aromatic compounds, genetics

## Abstract

Aromatic compounds are an important source of commodity chemicals traditionally produced from fossil fuels. Aromatics derived from plant lignin can potentially be converted into commodity chemicals through depolymerization followed by microbial funneling of monomers and low molecular weight oligomers. This study investigates the catabolism of the β-5 linked aromatic dimer dehydrodiconiferyl alcohol (DC-A) by the bacterium *Novosphingobium aromaticivorans*. We used genome-wide screens to identify candidate genes involved in DC-A catabolism. Subsequent *in vivo* and *in vitro* analyses of these candidate genes elucidated a catabolic pathway composed of four required gene products and several partially redundant dehydrogenases that convert DC-A to aromatic monomers that can be funneled into the central aromatic metabolic pathway of *N. aromaticivorans*. Specifically, a newly identified γ-formaldehyde lyase, PcfL, opens the phenylcoumaran ring to form a stilbene and formaldehyde. A lignostilbene dioxygenase, LsdD, then cleaves the stilbene to generate the aromatic monomers vanillin and 5-formylferulate (5-FF). We also showed that the aldehyde dehydrogenase FerD oxidizes 5-FF before it is decarboxylated by LigW, yielding ferulic acid. We found that some enzymes involved in the β-5 catabolism pathway can act on multiple substrates and that some steps in the pathway can be mediated by multiple enzymes, providing new insights into the robust flexibility of aromatic catabolism in *N. aromaticivorans*. A comparative genomic analysis predicted that the newly discovered β-5 aromatic catabolic pathway is common within the order Sphingomonadales.

**IMPORTANCE:**

In the transition to a circular bioeconomy, the plant polymer lignin holds promise as a renewable source of industrially important aromatic chemicals. However, since lignin contains aromatic subunits joined by various chemical linkages, producing single chemical products from this polymer can be challenging. One strategy to overcome this challenge is using microbes to funnel a mixture of lignin-derived aromatics into target chemical products. This approach requires strategies to cleave the major inter-unit linkages of lignin to release monomers for funneling into valuable products. In this study, we report newly discovered aspects of a pathway by which the *Novosphingobium aromaticivorans* DSM12444 catabolizes aromatics joined by the second most common inter-unit linkage in lignin, the β-5 linkage. This work advances our knowledge of aromatic catabolic pathways, laying the groundwork for future metabolic engineering of this and other microbes for optimized conversion of lignin into products.

## INTRODUCTION

Over the past century, aromatic compounds have proven integral to industries that generate critical chemicals and materials for society. For example, aromatic compounds are precursors for the production of plastics, adhesives, medicinal compounds, and flavorings. Most of today’s industrial aromatics are derived from fossil fuels. However, there is increasing interest in identifying renewable raw materials that can serve as alternative sources of these valuable chemicals.

**Fig 1:**
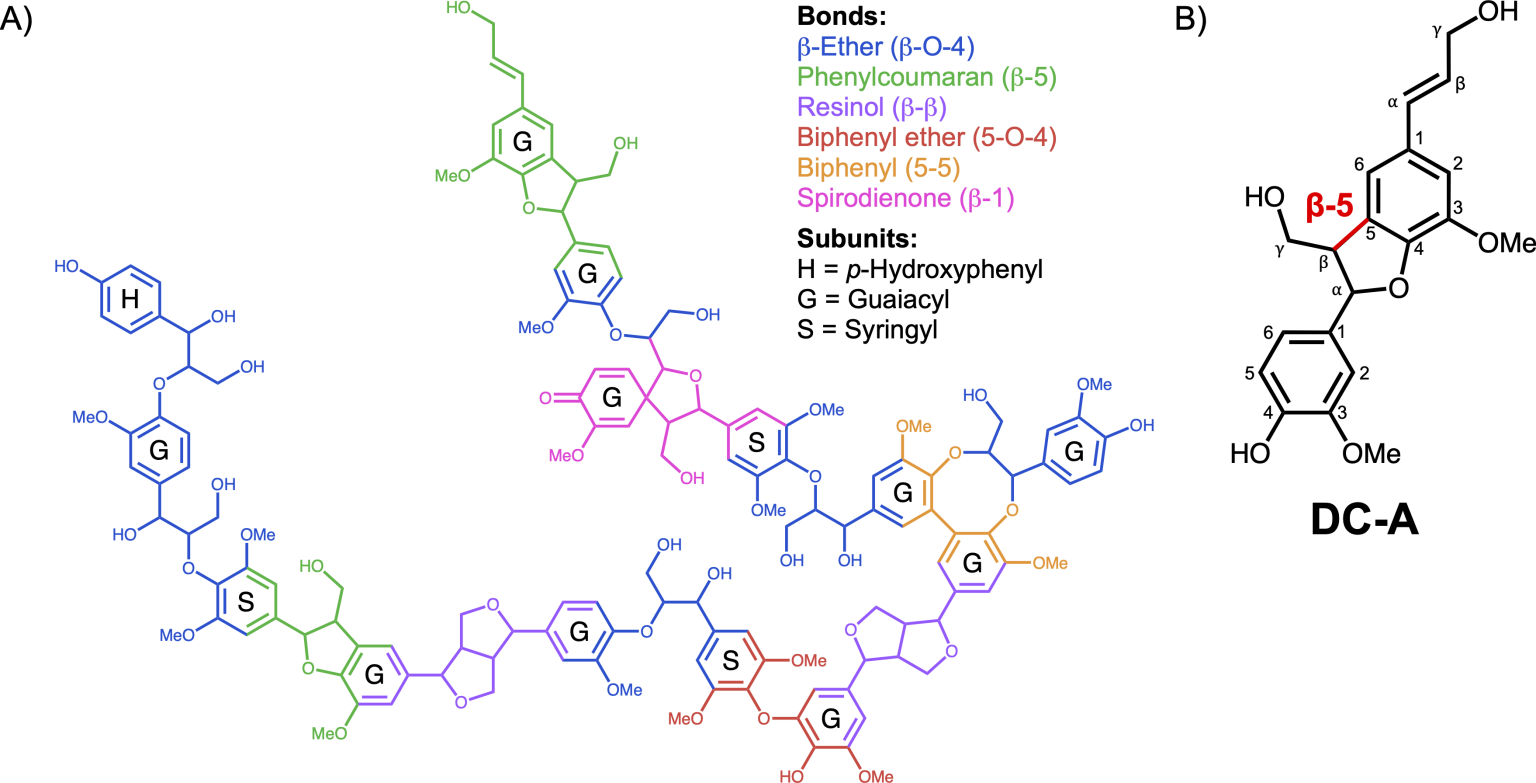
DC-A models β-5 linked lignin aromatics. (**A**) Model lignin polymer that illustrates major interunit linkages and aromatic subunits. (**B**) Structure of dehydrodiconiferyl alcohol (DC-A), a β-5 linked aromatic dimer composed of two G-family aromatic subunits. The β-5 bond is highlighted in red.

The plant polymer lignin can comprise up to 40% of the dry weight of plant biomass, making it the second most abundant biopolymer on the planet (1) and an attractive source of renewable aromatics for producing chemicals. Lignin is a heteropolymer composed of syringyl (S), guaiacyl (G), and *p*-hydroxyphenyl (H) aromatic subunits which differ in the number of methoxy groups attached to the aromatic ring (two, one, or zero, respectively) (2, 3). Since lignin polymers are synthesized via radical chemistry in plants, the aromatic subunits are joined by a variety of interunit bonds (Fig. 1A) (4–6). The chemical heterogeneity of its inter-aromatic linkages makes lignin recalcitrant to deconstruction, so it has traditionally been burned for fuel (1, 7, 8). However, strategies are emerging to convert the aromatic subunits of lignin to commodity chemicals and materials that are needed by society (2, 8).

One promising strategy is to use the aromatic compounds resulting from depolymerization of lignin as carbon sources that microbes can funnel into valuable products (9–12). *Novosphingobium aromaticivorans* DSM12444 is an Alphaproteobacterium with properties that make it a potential microbial chassis for lignin valorization. *N. aromaticivorans* can metabolize a variety of natural and chemically modified aromatic monomers and oligomers and it can co-metabolize aromatic compounds with other carbon sources (13, 14). In addition, native metabolic pathways enable engineered strains of this bacterium to funnel the products of depolymerized lignin into commodity chemicals such as 2-pyrone-4,6-dicarboxylic acid (PDC) (10, 15), *cis-cis-*muconic acid (16), and carotenoids (17). This study uses a previously engineered strain of *N. aromaticivorans* (12444PDC), in which *ligI*, *desC*, and *desD* have been deleted so that it converts S-, G-, and H-aromatics into PDC (10), which is a potential platform chemical for industrial valorization (18, 19).

While metabolic pathways by which *N. aromaticivorans* funnels aromatic monomers into central aromatic metabolism have been characterized (10, 20, 21), less is known about how it catabolizes aromatics joined by the various interunit bonds present in lignin. To date, only the pathways for catabolism of the most abundant interunit bond, the β-O-4 linkage (22, 23), as well as the β-1 linkage (24), have been elucidated in *N. aromaticivorans*. Catabolic pathways for aromatic oligomers containing other abundant interunit linkages have been reported in some organisms, but knowledge gaps remain in the pathways used by this bacterium.

This work sought to investigate the ability of *N. aromaticivorans* to catabolize β-5 (phenylcoumaran) linked aromatics. β-5 linked aromatics represent the second most abundant interunit linkage in lignin, accounting for up to 12% of the total interunit bonds depending on the biomass source (25, 26). The only pathway for the catabolism of β-5 linked aromatics has been proposed in *Sphingomonas paucimobilis* TMY10009 (27) and characterized in *Sphingobium* sp. SYK-6 (28–32), while one enzyme with activity on β-5 linked aromatics has been identified in *Agrobacterium* sp. (33). However, there are reports of significant differences in either the ability to catabolize aromatic compounds or the enzymes involved in the catabolic pathways of members of the order Sphingomonadales (11, 12, 20). Thus, it is important to identify similarities and differences in aromatic catabolism among different bacteria when developing strategies to valorize lignin.

The goal of this study was to determine whether and how *N. aromaticivorans* catabolizes aromatics joined by a β-5 linkage. To do this, we synthesized dehydrodiconiferyl alcohol (DC-A), a dimer composed of two G-aromatic monomers connected by a β-5 interunit linkage (Fig. 1B). We found that *N. aromaticivorans* can grow on DC-A and funnel it through its central aromatic metabolism. We combined data from two genome-wide screens to identify candidate genes involved in DC-A catabolism, followed by *in vivo* analysis of defined mutants and *in vitro* enzyme activity assays to test the roles of candidate genes and proteins in catabolism of this β-5 linked aromatic dimer. This approach defined a pathway for *N. aromaticivorans* DC-A catabolism that contains enzymes not previously known to be involved in aromatic dimer catabolism. Furthermore, comparative genomic analysis allowed us to predict that gene products involved in this catabolic pathway are widespread among the order Sphingomonadales.

## RESULTS

### *N. aromaticivorans* catabolizes DC-A

**Fig 2:**
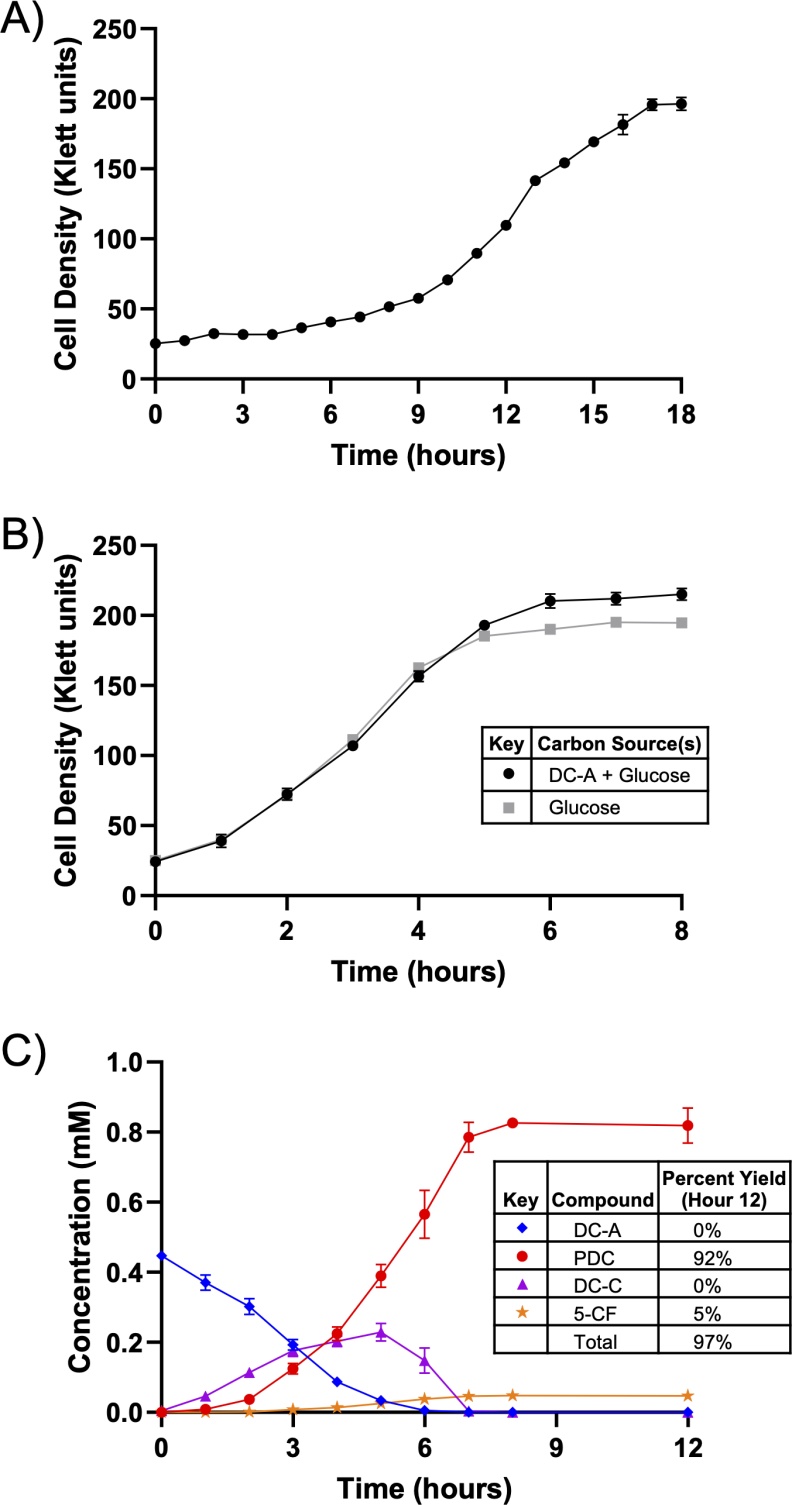
*N. aromaticivorans* funnels DC-A into central aromatic metabolism. (**A**) Growth of WT *N. aromaticivorans* in SMB minimal medium with DC-A as the sole carbon source. (**B**) Growth of 12444PDC in SMB minimal medium containing either DC-A plus glucose or glucose alone as carbon sources. (**C**) Metabolite concentrations in the extracellular medium of 12444PDC grown in SMB minimal medium with DC-A plus glucose as carbon sources. Error bars represent standard deviation across biological triplicates.

To test whether *N. aromaticivorans* can catabolize the β-5 linked dimer DC-A, we used a *sacB-* strain (23) as the wild type (WT) and grew it in standard mineral base (SMB) minimal medium with DC-A as the sole carbon source. We found that WT *N. aromaticivorans* grows on DC-A under these conditions (Fig. 2A). This led us to predict that the *N. aromaticivorans* genome encodes enzymes that cleave the β-5 linkage and metabolize the resulting G-family aromatic monomers.

We then asked whether *N. aromaticivorans* funnels these monomers through the known central aromatic metabolic pathway. To answer this question, we took advantage of the properties of *N. aromaticivorans* strain 12444PDC, which contains mutations in the central aromatic catabolic pathway that allow it to produce PDC when grown in the presence of many G-family aromatics (10). However, since G-aromatics are funneled into PDC in this strain, glucose or another alternative carbon source is required for growth. 12444PDC grown in the presence of 1 g/L glucose and 0.4 mM DC-A grows at a similar rate but to a slightly higher density than when it uses glucose as a sole carbon source (Fig. 2B), suggesting that both the glucose and some of the DC-A are used to produce biomass.

We used high-pressure liquid chromatography-mass spectrometry (HPLC-MS) to analyze the culture medium of 12444PDC grown in the presence of DC-A and glucose for consumption of DC-A and accumulation of PDC or other aromatic intermediates (see Fig. 4 for chemical structures). We found that DC-A disappears from the culture medium and PDC accumulates at 92% of the expected yield, assuming that one mole of DC-A would generate two moles of PDC (Fig. 2C). We used HPLC-MS to identify unknown aromatics (Table S1), including 5-carboxyferulate (5-CF), which represents 5% of the aromatics present in the medium at the end of the incubation period (Fig. 2C). We also observed the transient extracellular accumulation of trace amounts of a compound that was subsequently identified as dehydrodiconiferyl aldehyde (DC-L) (File S1) and the accumulation of a compound identified as dehydrodiconiferyl carboxylic acid (DC-C), suggesting the side chain of DC-A is oxidized from an alcohol to an aldehyde and then to a carboxylic acid. These results led us to conclude that *N. aromaticivorans* can funnel both G-family monomers of the β-5 linked DC-A dimer through its central aromatic metabolic pathway.

### Genome-wide screens identify candidate genes involved in DC-A catabolism

**Fig 3:**
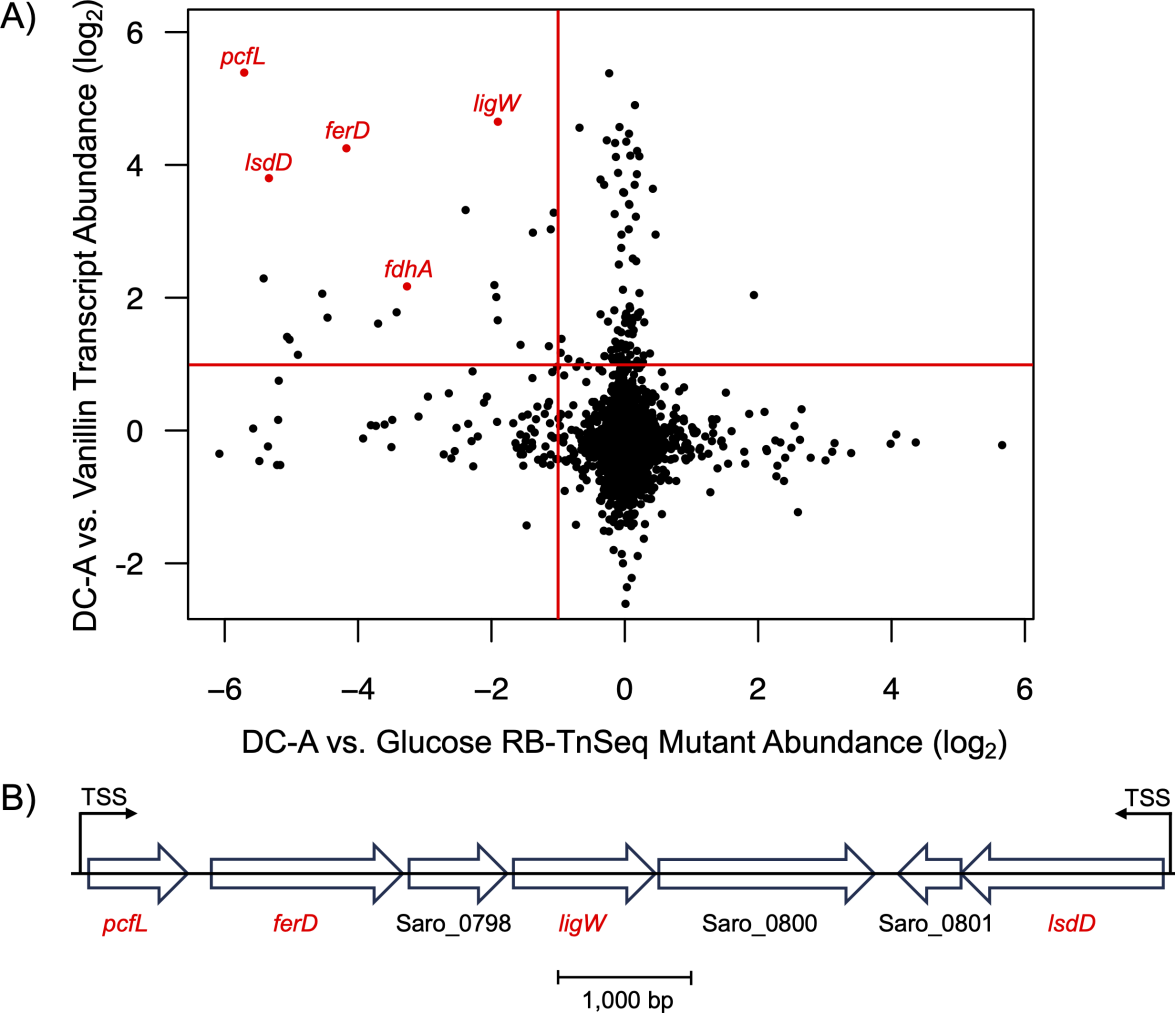
Genome-wide screens identify candidate genes for DC-A catabolism. (**A**) Dot plot (log_2_ scale) of RNA-Seq (y-axis) and RB-TnSeq (x-axis) data sets, with each dot representing a single gene. The horizontal and vertical red lines mark a two-fold increase in transcript abundance when *N. aromaticivorans* PDC12444 is grown on DC-A compared to vanillin and a two-fold abundance reduction of a disrupted gene when a *N. aromaticivorans* DSM12444 RB-TnSeq library is grown on DC-A compared to glucose, respectively. The five candidate genes investigated in this study are labeled in red. (**B**) The genomic region containing four of the five candidate genes. Candidate genes are labeled in red. Experimentally determined transcription start sites (TSS) are labeled (34).

Based on the above results, we sought to identify potential gene products involved in the catabolic pathway for β-5 linked aromatics in *N. aromaticivorans*. To do this, we integrated data from a pair of genome-wide screens. In one approach, we used RNA-Seq to compare mid-log phase transcript abundances of *N. aromaticivorans* 12444PDC grown on glucose plus either DC-A or the G-family aromatic monomer vanillin, which was used as a control because we predicted this aromatic monomer to be a product of DC-A catabolism that is further metabolized by known pathways (20, 21). We focused on the 126 transcripts that exhibited a greater than two-fold, statistically significant increase in abundance when grown in the presence of DC-A compared to cells grown in the presence of vanillin (Fig. 3A). Addition, we performed RNA-Seq experiments using glucose alone (Fig. S2) and glucose plus the G-family monomer ferulic acid (Fig. S2) as controls, which yielded similar results.

In a second genome-wide screen, we used an existing *N. aromaticivorans* randomly barcoded transposon insertion sequencing (RB-TnSeq) library (21) to identify insertions that led to fitness defects when cells were grown on DC-A as a sole carbon source compared to those grown on glucose alone. In this screen, we found 91 genes for which transposon insertions led to a greater than two-fold reduced abundance (>50% fitness decrease) after ~6.5 doublings when using DC-A compared to glucose as sole carbon sources (Fig. 3A).

Of the 91 transposon insertions that met the two-fold abundance reduction threshold in the RB-TnSeq screen, 22 were also among the candidates from the DC-A vs. vanillin RNA-Seq screen. Subsequent analysis centered on five candidate genes annotated as encoding proteins with predicted enzymatic activity (Table S2). Four of these five genes are found in two adjacent predicted transcription units (Fig. 3B), leading us to hypothesize that the gene products encoded by this region of the genome play a key role in DC-A catabolism.

**Fig 4:**
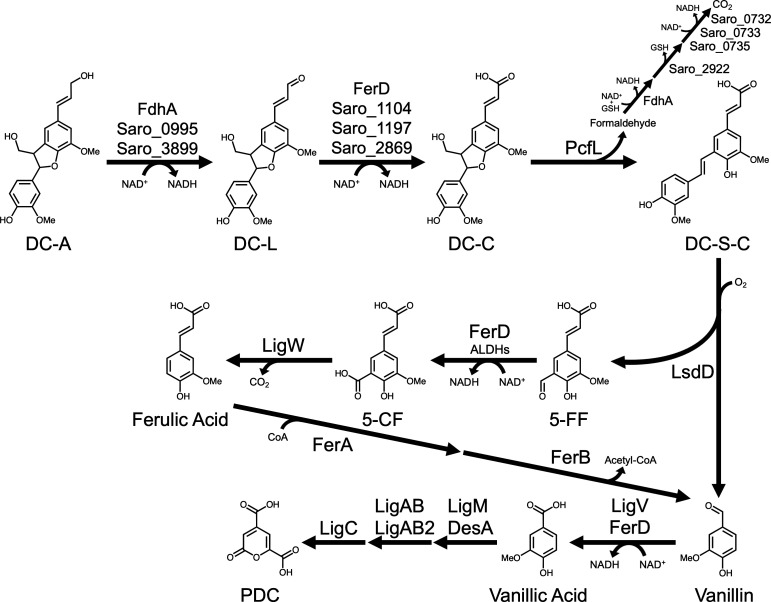
Proposed catabolic pathway for DC-A in *N. aromaticivorans*. The allylic alcohol side chain of DC-A is oxidized to DC-L and then to DC-C by dehydrogenases. The five-member ring of DC-C is opened by PcfL to form DC-S-C, which is then cleaved by LsdD into vanillin and 5-FF. 5-FF is oxidized to 5-CF by FerD and other dehydrogenases before it is decarboxylated by LigW to form ferulic acid. Metabolism of ferulic acid and vanillin to PDC by *N. aromaticivorans* has been previously described (10, 21). The gene products predicted to be involved in the metabolism of formaldehyde following oxidation by FdhA are based on the homology of *N. aromaticivorans* gene products with known S-glutathione hydrolases (Saro_2822) (35) and the subunits of a formate dehydrogenase complex (Saro_0732, Saro_0733, and Saro_0735) (36).

Below, we present data from *in vivo* and *in vitro* experiments used to test this hypothesis. Combined, the data from these experiments identify dehydrogenases that can oxidize the allylic side chain of DC-A in a stepwise manner as well as gene products that open the phenylcoumaran ring in the β-5 interunit linkage of DC-C, cleave the resulting dehydrodiconiferyl stilbene carboxylic acid (DC-S-C), and funnel the monomeric G-family cleavage product 5-formyl ferulate (5-FF) into the *N. aromaticivorans* central aromatic metabolic pathway (Fig. 4).

### PcfL opens the DC-A phenylcoumaran ring

**Fig 5:**
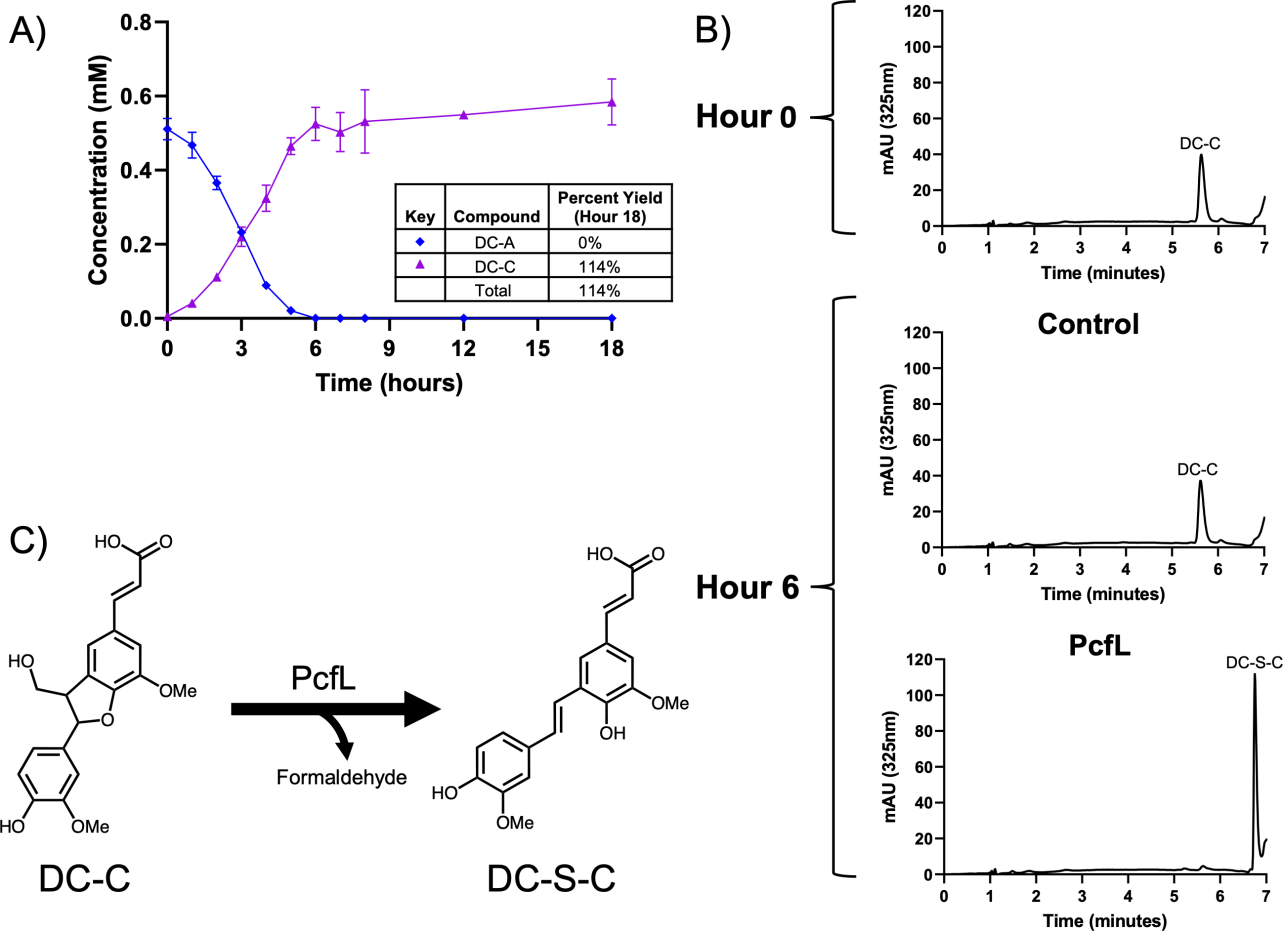
PcfL converts DC-C to DC-S-C. (**A**) Metabolite concentrations in the extracellular medium of 12444PDCΔ*pcfL* grown in SMB minimal medium with DC-A plus glucose as carbon sources. Error bars represent standard deviation across biological triplicates. (**B**) Representative HPLC chromatograms of *in vitro* reactions containing DC-C and either control *E. coli* B834 cell extract or cell extract from *E. coli* B834 expressing recombinant PcfL. (**C**) Conversion of DC-C to DC-S-C by PcfL.

We examined the role of PcfL (Saro_0796) in DC-A catabolism by comparing metabolism of this β-5 linked aromatic dimer in the 12444PDC strain with a Δ*pcfL* in-frame deletion strain (12444PDCΔ*pcfL*). We found that DC-A disappears from the growth medium of this mutant (Fig. 5A), but unlike the parent strain (Fig. 2C), it does not accumulate PDC. Instead, when grown in the presence of DC-A and glucose, 12444PDCΔ*pcfL* accumulates a compound which we were able to identify as DC-C using a synthetic DC-C standard. In addition, when we quantified DC-C in the 12444PDCΔ*pcfL* medium, we found that one mole of DC-C accumulates per mole of DC-A. Since DC-A catabolism does not progress past DC-C in cells that lack *pcfL*, we proposed that DC-C is a substrate for this enzyme.

To evaluate this hypothesis, we incubated *E. coli* cell extracts containing a recombinant PcfL enzyme with pure DC-C. We found that PcfL-containing cell extract converts DC-C to another compound that matches synthetic DC-S-C, while a control extract exhibits no detectable conversion of DC-C under the same conditions (Fig. 5B). Based on these data and the 44% amino acid identity between PcfL and the γ-formaldehyde lyase LdpA that contributes to β-1 linked aromatic catabolism in *N. aromaticivorans* (24, 37), we proposed that PcfL removes formaldehyde from DC-C to form the stilbene DC-S-C. We further predicted that the formaldehyde released during this reaction is oxidized by the putative glutathione-dependent dehydrogenase Saro_0874, which we named FdhA (formaldehyde dehydrogenase A), based on homology with an enzyme found in *Rhodobacter sphaeroides* (38, 39). Upon testing these hypotheses, we found that PcfL produces formaldehyde from DC-C *in vitro* (Fig. S3) and that a 12444PDCΔ*fdhA* mutant accumulates more extracellular formaldehyde than the parent strain when grown in the presence of DC-A and glucose (Fig. S4A and B). In sum, our data indicate that PcfL is a newly identified γ-formaldehyde lyase that deformylates DC-C, yielding DC-S-C and formaldehyde (Fig. 5C). Based on these results, we named this gene product PcfL to denote its activity as a phenylcoumaran γ-formaldehyde lyase.

### LsdD cleaves DC-S-C into two aromatic monomers

Our results suggest that *N. aromaticivorans* contains one or more gene products that use the stilbene DC-S-C as a substrate. LsdD (Saro_0802) is a candidate for cleavage of DC-S-C since this gene product shares 80% amino acid identity with the S*phingobium* sp. SYK-6 enzyme LsdD, which has been reported to convert DC-S-C into vanillin and 5-FF (30). Furthermore, *N. aromaticivorans* LsdD (named NOV1 in other work) is an iron-dependent dioxygenase that is known to cleave stilbenes such as resveratrol *in vitro* (40, 41).

**Fig 6:**
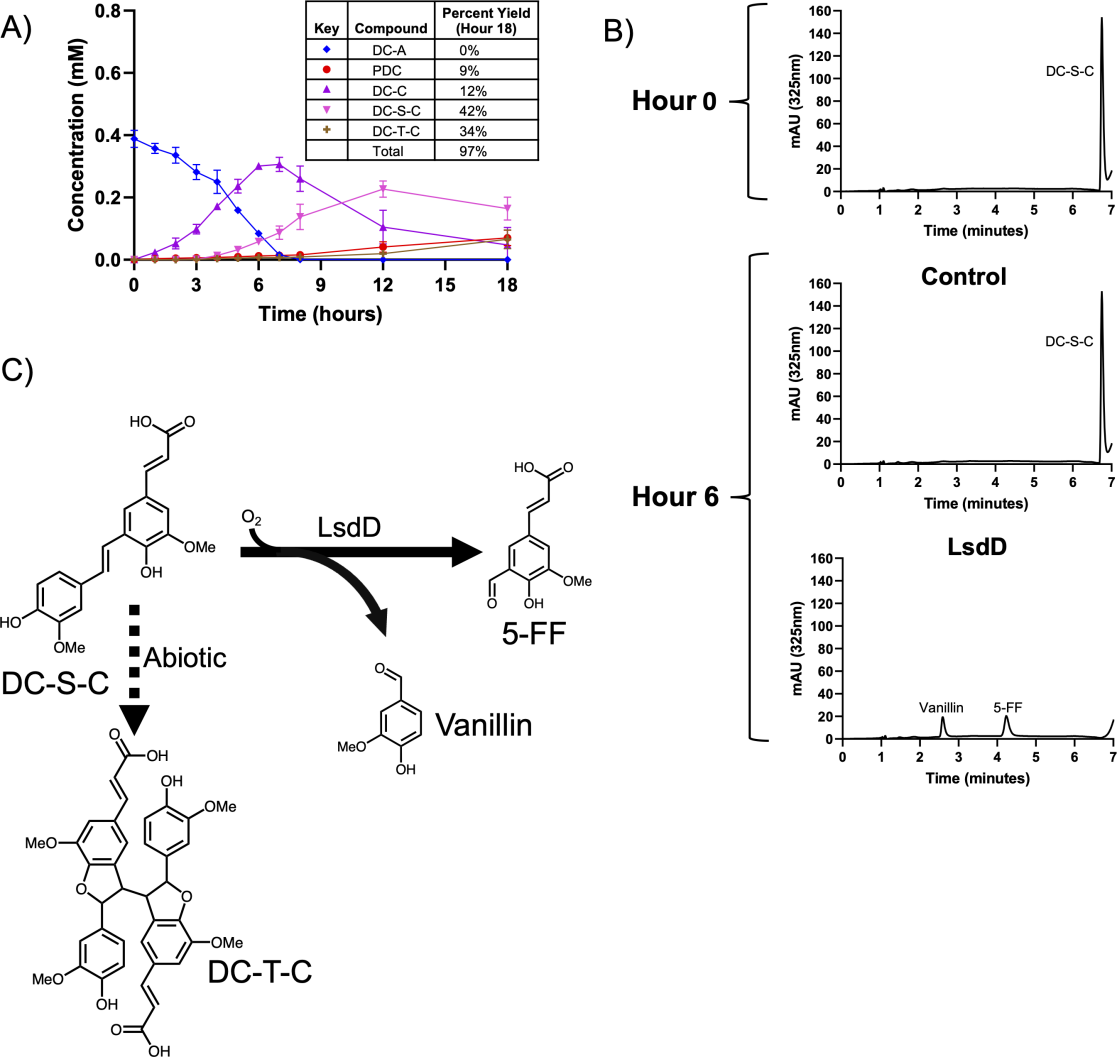
LsdD cleaves DC-S-C to form 5-FF and vanillin. (**A**) Metabolite concentrations in the extracellular medium of 12444PDCΔ*lsdD* grown in SMB minimal medium with DC-A plus glucose as carbon sources. Error bars represent standard deviation across biological triplicates. (**B**) Representative HPLC chromatograms of *in vitro* reactions containing DC-S-C and either control *E. coli* cell extract or cell extract from *E. coli* expressing recombinant LsdD. (**C**) Cleavage of DC-S-C to 5-FF and vanillin by LsdD and abiotic dimerization of DC-S-C to DC-T-C.

As predicted by this hypothesis, we found that 12444PDCΔ*lsdD* grown in the presence of DC-A and glucose accumulates DC-S-C in the medium (Fig. 6A). This strain also accumulates more DC-C than the parent strain (Fig. 2B) before it is metabolized to DC-S-C, with a detectable amount of DC-C still present in the medium after the 18-hour incubation. In addition, HPLC-MS analysis of extracellular compounds in the 12444PDCΔ*lsdD* strain medium indicated the presence of another unknown aromatic compound. In control experiments, we found that DC-S-C is subject to abiotic homodimerization to form the dehydroconiferyl tetramer carboxylic acid DC-T-C when incubated in SMB minimal medium (Fig. S5A and B). At the end of the incubation, 76% of the extracellular aromatics produced from DC-A by 12444PDCΔ*lsdD* are found in the sum of DC-S-C and DC-T-C, while only 9% are converted into PDC. We propose that the low amount of PDC excreted by this strain is derived from the activity of one or more enzymes besides LsdD in cleaving DC-S-C (see Discussion).

We tested the predicted activity of LsdD by incubating *E. coli* cell extracts containing a recombinant LsdD enzyme with synthetic DC-S-C. When incubated with DC-S-C in the absence of any cofactors, LsdD converts this substrate to 5-FF and vanillin (Fig. 6B). Therefore, we concluded that LsdD cleaves the β-5 linked stilbene DC-S-C into two G-family monomers (Fig. 6C) that can then be funneled into the central pathway for aromatic metabolism.

### FerD and LigW convert 5-FF to ferulic acid

Our data indicate that the two monomeric products of DC-A catabolism are the G-aromatic monomers vanillin and 5-FF. In *N. aromaticivorans*, vanillin is known to be oxidized to vanillic acid by LigV before entering central G-aromatic metabolism (21). However, the enzymes that metabolize 5-FF have not been identified in this organism. Based on the data from our genome-wide screens, we hypothesized that the putative pyridine nucleotide-dependent ALDH FerD (Saro_0797) oxidizes 5-FF to 5-CF, which is then decarboxylated by LigW (Saro_0799) to form ferulic acid. Ferulic acid is known to be converted into vanillin via a previously described pathway in *N. aromaticivorans* (21).

**Fig 7:**
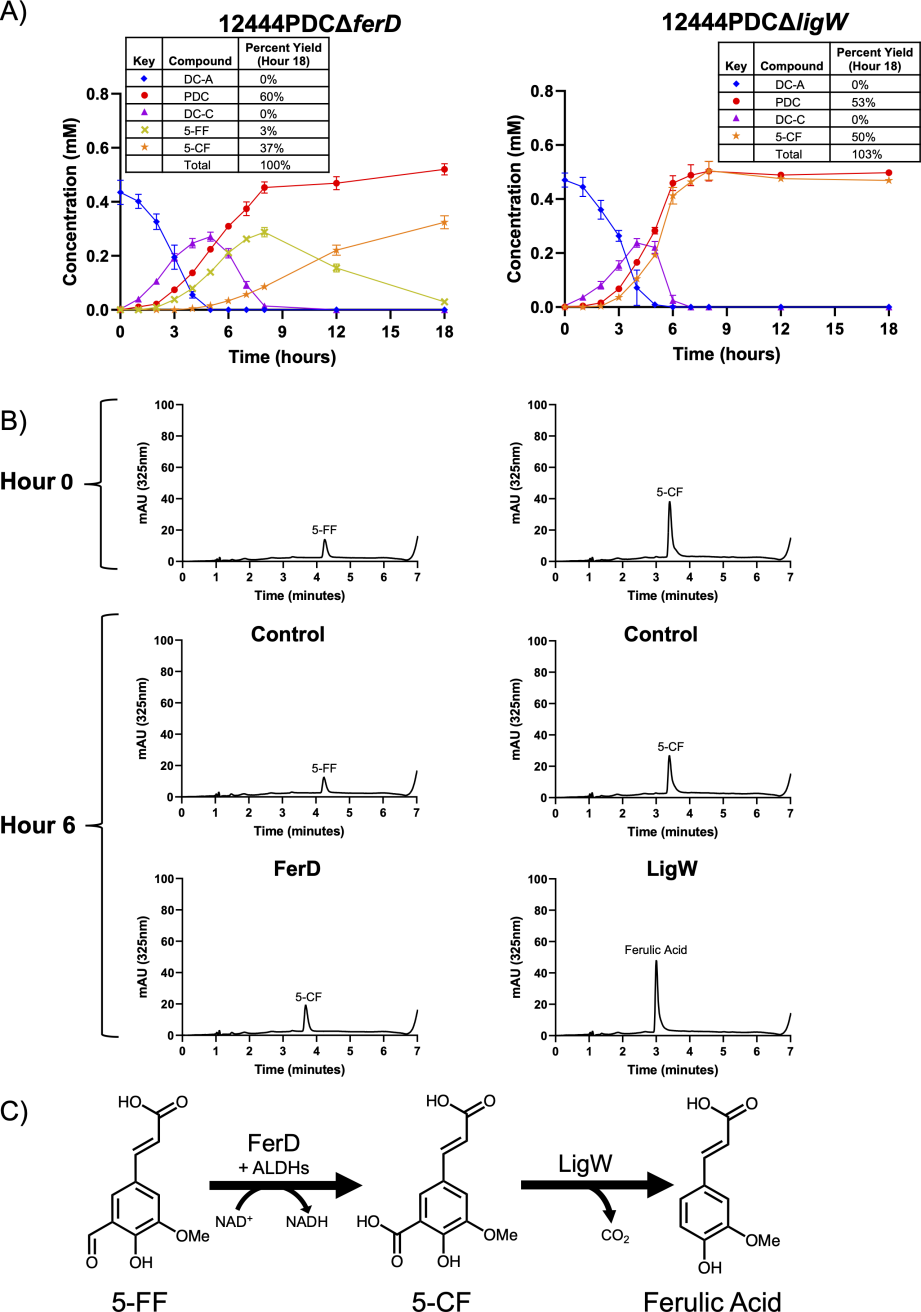
FerD and LigW convert 5-FF to 5-CF and then ferulic acid. (**A**) Metabolite concentrations in the extracellular medium of 12444PDCΔ*ferD* and 12444PDCΔ*ligW* grown in SMB minimal medium with DC-A plus glucose as carbon sources. Error bars represent standard deviation across biological triplicates. (**B**) Representative HPLC chromatograms of *in vitro* reactions (left) containing 5-FF plus NAD^+^ and either control *E. coli* B834 cell extract or cell extract of *E. coli* B834 expressing recombinant FerD or reactions (right) containing 5-CF and either control *E. coli* B834 cell extract or cell extract of *E. coli* B834 expressing recombinant LigW. (**C**) Oxidation of 5-FF to 5-CF by FerD and decarboxylation of 5-CF to ferulic acid by LigW.

Since the conversion of 5-FF to 5-CF occurs after DC-S-C cleavage, we predicted that growing 12444PDCΔ*ferD* in the presence of DC-A and glucose would result in the accumulation of one mole of both 5-FF and PDC per mole of DC-A. We found that 12444PDCΔ*ferD* cells transiently accumulate 5-FF in the medium. However, at later time points, as the concentration of 5-FF decreases, the concentration of 5-CF increases. 5-CF can then be funneled into PDC production, leading to the accumulation of 1.17 moles of PDC per mole of DC-A by the end of the incubation (Fig. 7A). To explain these results, we hypothesize that one or more other *N. aromaticivorans* dehydrogenases can oxidize 5-FF to 5-CF, albeit at a slower rate than FerD. In addition, *E. coli* cell extract containing recombinant FerD converts 5-FF into 5-CF (Fig. 7B). As expected, FerD-containing cell extract requires NAD^+^ to convert 5-FF to 5-CF (Fig. S6A) and a purified recombinant FerD protein reduces NAD^+^ to NADH during this reaction (Fig. S6B). From these data, we propose that the NAD^+^-dependent dehydrogenase FerD is the major gene product responsible for 5-FF to 5-CF conversion (Fig. 7C) when cells are grown on DC-A, but that other yet uncharacterized enzymes can also catalyze this reaction.

We investigated the predicted role of LigW in decarboxylation of 5-CF to ferulic acid by growing a 12444PDCΔ*ligW* strain in a medium containing DC-A and glucose. Under these conditions, we found that cells lacking *ligW* accumulate ~1 mole of both PDC and 5-CF per mole of DC-A (Fig. 7A), suggesting that this gene product is responsible for the decarboxylation of 5-CF. As predicted, we found that *E. coli* cell extracts expressing recombinant LigW can convert 5-CF into ferulic acid *in vitro* (Fig. 7B). We therefore concluded that LigW decarboxylates 5-CF in *N. aromaticivorans* (Fig. 7C).

### Multiple dehydrogenases can oxidize the DC-A allylic alcohol side chain

Given the predicted intermediates of DC-A catabolism (Fig. 4), we hypothesized that *N. aromaticivorans* contains enzymes that oxidize the allylic alcohol to an aldehyde and then to a carboxylic acid. The only proteins annotated as either alcohol dehydrogenases (ADH) or aldehyde dehydrogenases (ALDH) that were identified as candidates in our genome-wide screens were FdhA and FerD, respectively. However, in the 12444PDCΔ*ferD* and 12444PDCΔ*fdhA* strains, the DC-A allylic side chain was still oxidized to a carboxylic acid (Fig. 7A; Fig. S4A). Based on these findings, we hypothesized that *N. aromaticivorans* contains multiple partially redundant ADHs and ALDHs that convert DC-A to DC-L and DC-L to DC-C.

We tested this hypothesis by analyzing the activity of eight putative ADHs and nine putative ALDHs for which transcripts represented >2% of the total RNA coding for ADHs or ALDHs when *N. aromaticivorans* is grown in the presence of DC-A (Table S3). We performed enzyme assays to determine the activity of these gene products by expressing recombinant versions of the proteins in *E. coli* and incubating cell extracts normalized to the same protein concentration with either DC-A or DC-L with and without NAD^+^ (or PQQ for Saro_2870). We used differences in absorption spectra (Fig. S7) to monitor conversion from DC-A to DC-L and DC-L to DC-C. Control experiments show that none of the cell extracts containing recombinant ADHs or ALDHs were active on these substrates in the absence of NAD^+^.

**Fig 8:**
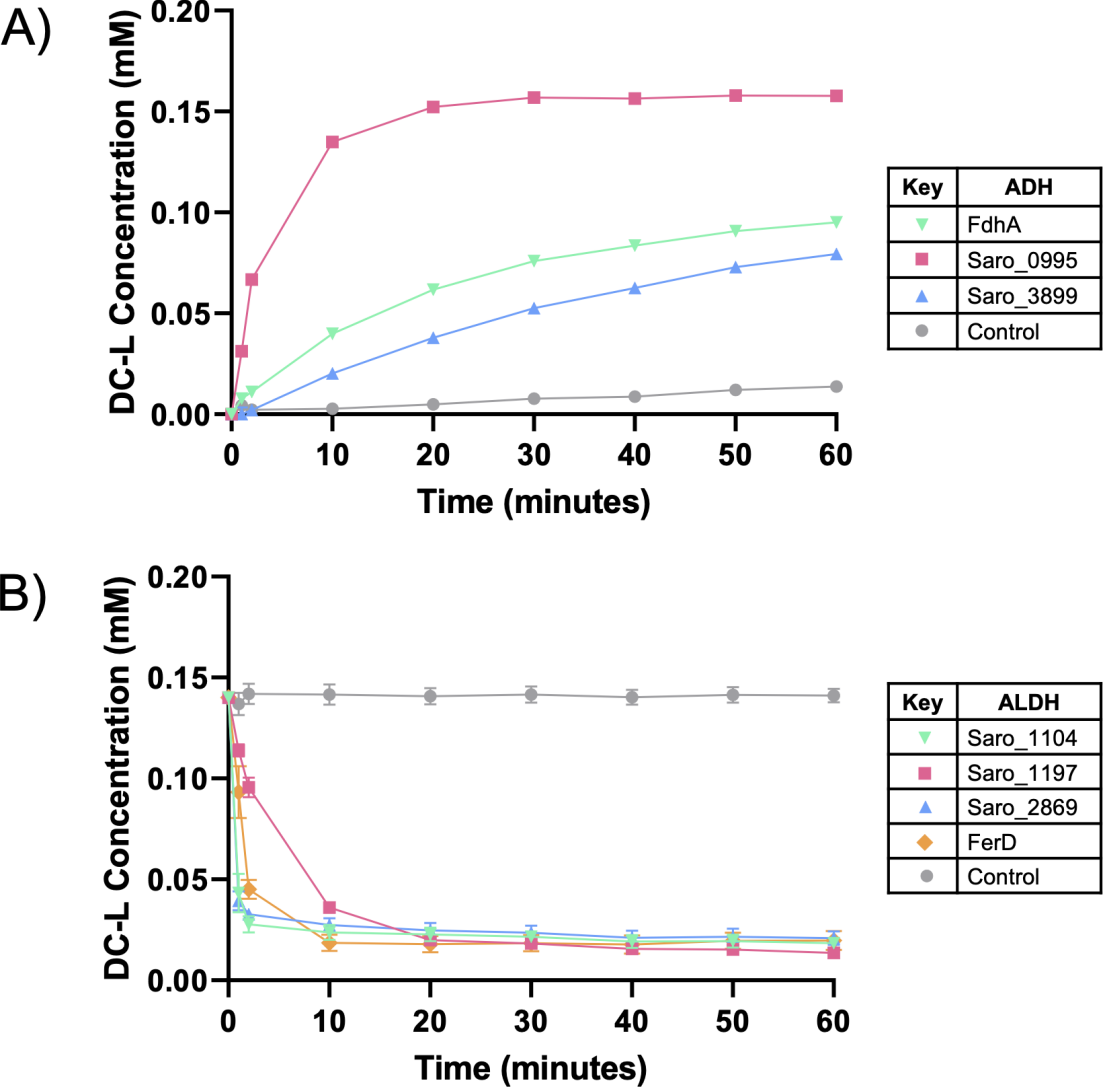
Multiple partially redundant ADHs and ALDHs can oxidize the allylic side chain of DC-A. The concentration of DC-L over 1 hour long *in vitro* assays containing (A) DC-A, NAD^+^, and a control *E. coli* B834 cell extract or cell extracts of *E. coli* B834 expressing recombinant candidate ADHs or (B) DC-L, NAD^+^, and control *E. coli* B834 cell extract or cell extracts of *E. coli* B834 expressing recombinant candidate ALDHs. For clarity of presentation, only dehydrogenases exhibiting activity on the tested substrates are shown. Error bars represent standard deviation across triplicates.

We found that the putative ADHs FdhA, Saro_0995, and Saro_3899 convert DC-A to DC-L *in vitro*, with Saro_0995 exhibiting the highest activity under our assay conditions (Fig. 8A). There was some conversion of DC-A to DC-L when a control *E. coli* extract was incubated with DC-A, suggesting that one or more native *E. coli* enzymes have limited activity on DC-A. However, the conversion of DC-A to DC-L was much faster when using extracts prepared from cells expressing the ADHs listed above.

Using the same approach, we found that the cell extracts containing recombinant versions of the putative ALDHs FerD, Saro_1104, Saro_1197, and Saro_2869 can convert DC-L to DC-C *in vitro* (Fig. 8B). The similar activity of extracts containing these ALDHs on DC-L suggests that they could each make a significant contribution to the metabolism of DC-L *in vivo*. Combined, the results of these experiments predict that multiple *N. aromaticivorans* enzymes can oxidize the DC-A allylic alcohol side chain to an aldehyde and then to a carboxylic acid.

### Reconstructing the DC-A catabolic pathway *in vitro*

**Fig 9:**
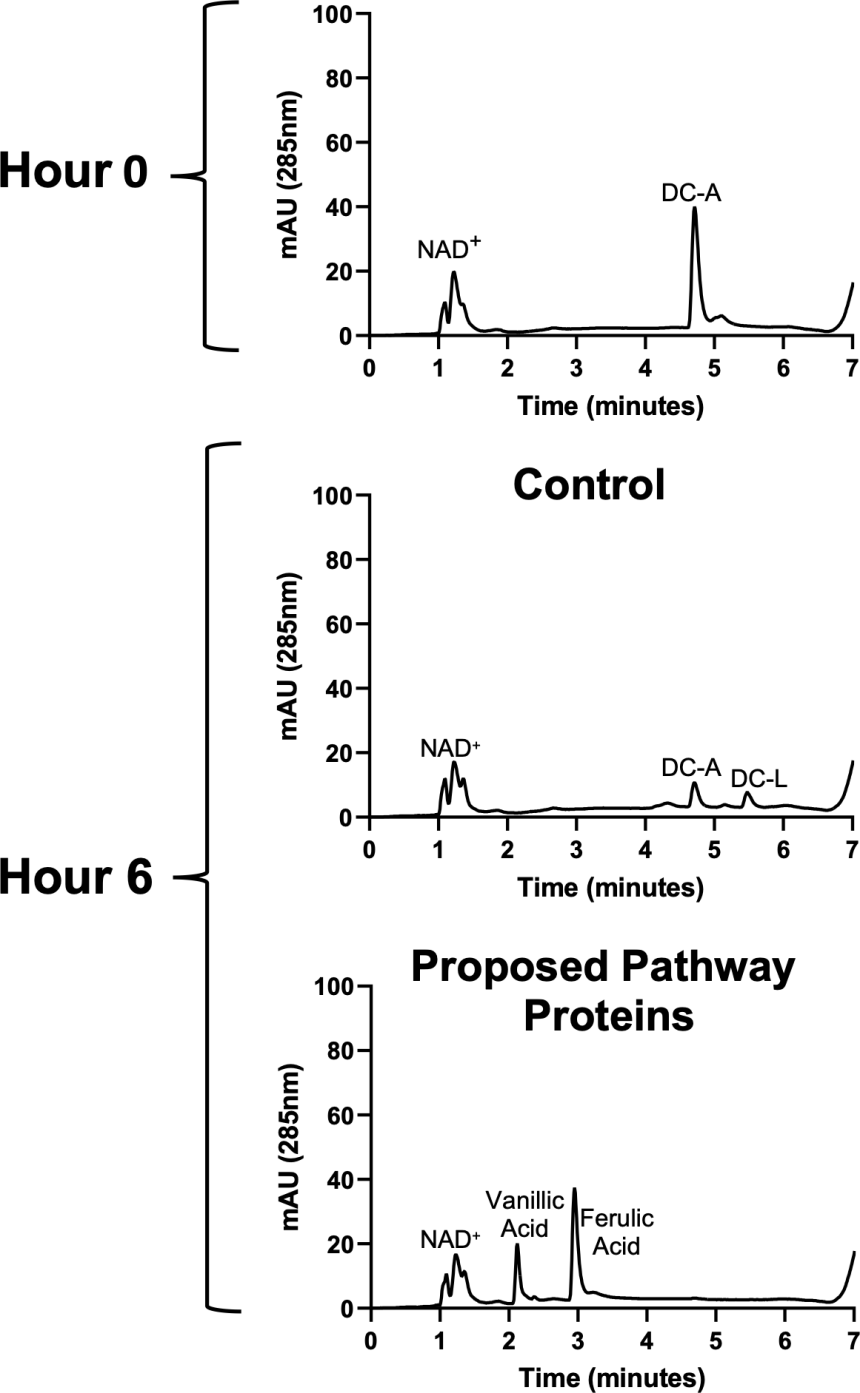
The proposed catabolic pathway enzymes can convert DC-A to ferulic acid and vanillic acid *in vitro*. Representative HPLC chromatograms of *in vitro* reactions containing DC-A plus NAD^+^ and either control *E. coli* B834 cell extract or cell extracts from *E. coli* B834 expressing recombinant Saro_0995, PcfL, LsdD, FerD, and LigW.

As an independent test of whether the enzymes described above are sufficient for the catabolism of DC-A to G-family aromatic monomers, we sought to reconstruct the entire *N. aromaticivorans* DC-A catabolic pathway *in vitro*. Based on the above results, we predicted that a mixture of cell extracts containing NAD^+^, the γ-formaldehyde lyase PcfL, the stilbene cleaving dioxygenase LsdD, the ALDH FerD, the decarboxylase LigW, and the ADH Saro_0995 would be able to convert DC-A to G-family aromatics. After incubating DC-A with these five cell extracts and NAD^+^, we observed complete conversion of DC-A to ferulic and vanillic acid (Fig. 9). When incubated with a control *E. coli* cell extract containing none of these *N. aromaticivorans* enzymes, ferulic acid and vanillic acid do not accumulate. However, DC-A is slowly converted to DC-L by the control extract, resulting in a mixture of DC-A and DC-L, in agreement with observations that some native *E. coli* enzymes have limited activity on DC-A (Fig. 8A). Overall, this experiment confirms that the *N. aromaticivorans* enzymes we identified are sufficient for the catabolism of DC-A to aromatic monomers that are funneled through known pathways into *N. aromaticivorans* central aromatic metabolism.

## DISCUSSION

Aromatic compounds are an important source of industrial products and there is increasing interest in renewable sources of these compounds. The abundant plant polymer lignin is a potential source of aromatics that could be used in the production of commodity chemicals. To valorize lignin, the various interunit linkages between aromatic subunits of this polymer must be cleaved, and the resulting mixture of monomers funneled into products (9, 10, 12). Recently, progress has been made in the biological funneling of aromatics into valuable chemicals using the Alphaproteobacterium *N. aromaticivorans* (15). In this study, we found that *N. aromaticivorans* contains enzymes capable of catabolizing aromatic dimers with β-5 linkages, which is the second most abundant interunit linkage in lignin (25, 26).

Specifically, we showed that *N. aromaticivorans* can grow on the model β-5 linked G-family aromatic dimer DC-A and that the engineered 12444PDC strain funnels both of its aromatic monomers into PDC production. By combining genomic, genetic, and biochemical assays, we identified gene products that are necessary and sufficient for catabolism of DC-A. Based on these studies, we proposed a catabolic pathway for the conversion of DC-A to intermediates in the known *N. aromaticivorans* central aromatic metabolic pathway.

### Oxidation of the DC-A allylic side chain

We identified enzymes that oxidize the allylic alcohol side chain of DC-A to an aldehyde and the aldehyde to a carboxylic acid. Our data show that three *N. aromaticivorans* pyridine nucleotide-dependent ADHs (FdhA, Saro_0995, and Saro_3899) can oxidize the allylic alcohol side chain of DC-A, producing the aldehyde DC-L. We also identified four pyridine nucleotide-dependent ALDHs (FerD, Saro_1104, Saro_1197, and Saro_2869) that can oxidize the aldehyde side chain of DC-L to generate the carboxylic acid DC-C. These findings are consistent with RNA-Seq and RB-TnSeq data that indicate increased transcript abundance for multiple ADHs and ALDHs but small or no fitness defects when these dehydrogenases are mutated, suggesting that oxidization of the allylic alcohol side chain of DC-A could be performed by multiple ADHs and ALDHs *in vivo* (Fig. 3A). Additional biochemical and genetic analyses would be needed to quantify the activity of each ADH and ALDH enzyme on DC-A or DC-L and their relative contribution to catabolism of these and other β-5 linked aromatics *in vivo*.

### Cleavage of the β-5 linkage

We found that the phenylcoumaran DC-C is converted to the stilbene DC-S-C and formaldehyde by the newly identified γ-formaldehyde lyase PcfL. This strategy for catabolism of a phenylcoumaran by *N. aromaticivorans* diverges from the one reported in another aromatic metabolizing member of the order Sphingomonadales, *Sphingobium* sp. SYK-6 (28, 29). In this bacterium, a pair of enantiospecific oxidoreductases, PhcC and PhcD, as well as other partially redundant dehydrogenases were shown to sequentially oxidize the phenylcoumaran alcohol to an aldehyde and then a carboxylic acid (28). Next, a pair of enantiospecific decarboxylases, PhcF and PhcG, decarboxylate and open the phenylcoumaran ring of DC-C to produce DC-S-C and CO_2_ (29). By comparison, the *N. aromaticivorans* pathway for generating a stilbene from DC-C requires only a single enzyme as PcfL opens the phenylcoumaran ring and releases formaldehyde in a single step. In addition, our finding that recombinant PcfL can completely convert DC-C into DC-S-C indicates that this enzyme is agnostic to the enantiomeric state of its substrate. An *Agrobacterium* sp. enzyme catalyzes a similar reaction in which it converts a phenylcoumaran to a stilbene, but this enzyme is a glutathione-dependent LigE family enzyme rather than a γ-formaldehyde lyase like PclF.

To our knowledge, the only homolog of PcfL that has been characterized is LdpA, which is another *N. aromaticivorans* gene product that converts a dimeric aromatic substrate into a stilbene and releases formaldehyde (24, 37). While we found that PcfL has activity with a phenylcoumaran substrate, LdpA acts on a diarylpropane dimer which is a reported intermediate in the *N. aromaticivorans* β-1 linked aromatic catabolic pathway (24). Since PcfL shares eight of the eleven active site residues of LdpA, future work should test if and how these amino acid differences contribute to the substrate preferences of these two enzymes.

Once DC-S-C forms, our data show this aromatic dimer is cleaved to form 5-FF and vanillin by the lignostilbene dioxygenase LsdD, a homolog of an enzyme previously reported in *Sphingobium* sp. SYK-6 (30). Cleavage of this β-5 linked stilbene by *N. aromaticivorans* mirrors the process in β-1 aromatic dimer metabolism, in which the stilbene produced by LdpA is then cleaved by the dioxygenase LsdA (also called NOV2). This combination of a γ-formaldehyde lyase followed by a lignostilbene dioxygenase is a newly described strategy for breaking both β-5 and β-1 interunit linkages in lignin.

### Funneling of monomers into central aromatic metabolism

Once the β-5 linked dimer DC-A is cleaved into monomeric products, vanillin and 5-FF are funneled into the *N. aromaticivorans* central G-aromatic metabolic pathway and can be converted into PDC. While vanillin is metabolized through a known pathway (21), our experiments identified enzymes involved in the conversion of 5-FF to 5-CF and then to ferulic acid. We found that 5-FF is oxidized to 5-CF by FerD with minor contributions from one or more uncharacterized ALDHs. We also found that LigW decarboxylates 5-CF to ferulic acid, which is metabolized to vanillin through a known pathway (21). A recently published analysis of 5-FF metabolism in *Sphingobium* sp. SYK-6 reports the same functions for FerD and LigW (31). *N. aromaticivorans* LigW has previously been shown to decarboxylate 5-carboxyvanillate (5-CV) (42), which contains a simple carboxylic acid in place of the allylic acid side chain of 5-CF. Thus, it appears that *N. aromaticivorans* LigW is a relatively broad specificity manganese-dependent aromatic decarboxylase that can function in the metabolism of both the β-5 linked aromatic catabolic pathway intermediate 5-CF and the predicted 5-5 linked aromatic catabolic pathway intermediate 5-CV (43).

### Redundant enzymes in the catabolism of β-5 linked aromatics

*N. aromaticivorans* is known to contain several enzymes with multiple functions in aromatic metabolism (20, 44), so it is not surprising for us to find that LigW is not the only enzyme in this pathway with activity on multiple aromatics. We also showed that the dehydrogenases FerD and FdhA display activity on multiple intermediates in the DC-A catabolic pathway. While FdhA is active in the conversion of DC-A to DC-L and in the catabolism of formaldehyde, FerD is a promiscuous ALDH that plays a crucial role in the oxidation of 5-FF to 5-CF but is also able to oxidize both DC-L to DC-C and vanillin to vanillic acid (Fig. S8).

In addition, PcfL deformylates not only DC-C but also DC-A and DC-L *in vitro* (Fig. S9A and B ), forming products that match the m/z of predicted allylic alcohol and allylic aldehyde stilbenes (Fig. S9C ). While we propose that side chain oxidation precedes conversion of the phenylcoumaran to a stilbene based on the transient accumulation of DC-C in the medium when 12444PDC is grown on DC-A (Fig. 2B), it is possible that PcfL converts some DC-A or DC-L to a stilbene prior to side chain oxidation (Fig. S10).

In addition to *N. aromaticivorans* enzymes acting on multiple aromatic substrates, it is known that multiple enzymes often mediate the same reaction in aromatic metabolism. Consistent with this, we found that allylic side chain oxidation of DC-A and oxidation of 5-FF are performed by multiple dehydrogenases. While our data indicate that LsdD plays a major role in the cleavage of DC-S-C into monomers, it is possible that one or both of two other *N. aromaticivorans* homologs of this dioxygenase , LsdA/NOV2 (Saro_2809) and Saro_3580, can also perform this reaction. Overall, our findings showcase the robust and flexible strategies *N. aromaticivorans* uses for funneling a range of aromatics into a central metabolic pathway.

### Conservation of β-5 linked aromatic catabolic pathways in the order Sphingomonadales

After uncovering the pathway for β-5 linked aromatic catabolism in *N. aromaticivorans*, we asked whether other organisms contain enzymes predicted to function in this pathway. To do so, we searched for homologs (>50% amino acid identity, >70% query coverage) of PcfL, LsdD, FerD, and LigW across all bacteria. We found that 82 organisms, all Alphaproteobacteria, are predicted to contain all four of these enzymes. Of those 82, all but *Maricaulis flavus* are members of the order Sphingomonadales. We also identified organisms with at least two homologs of β-5 linked aromatic catabolism enzymes, which are distributed across both gram-negative and gram-positive bacteria, including members of the orders Actinomyces, Gammaproteobacteria, Betaproteobacteria, and Bacilli (Fig. S11). Thus, we concluded that the complete *N. aromaticivorans* pathway for β-5 linked aromatics is almost exclusively found in Sphingomonadales, but that other bacteria are predicted to contain some of the enzymes described in this study.

**Fig 10:**
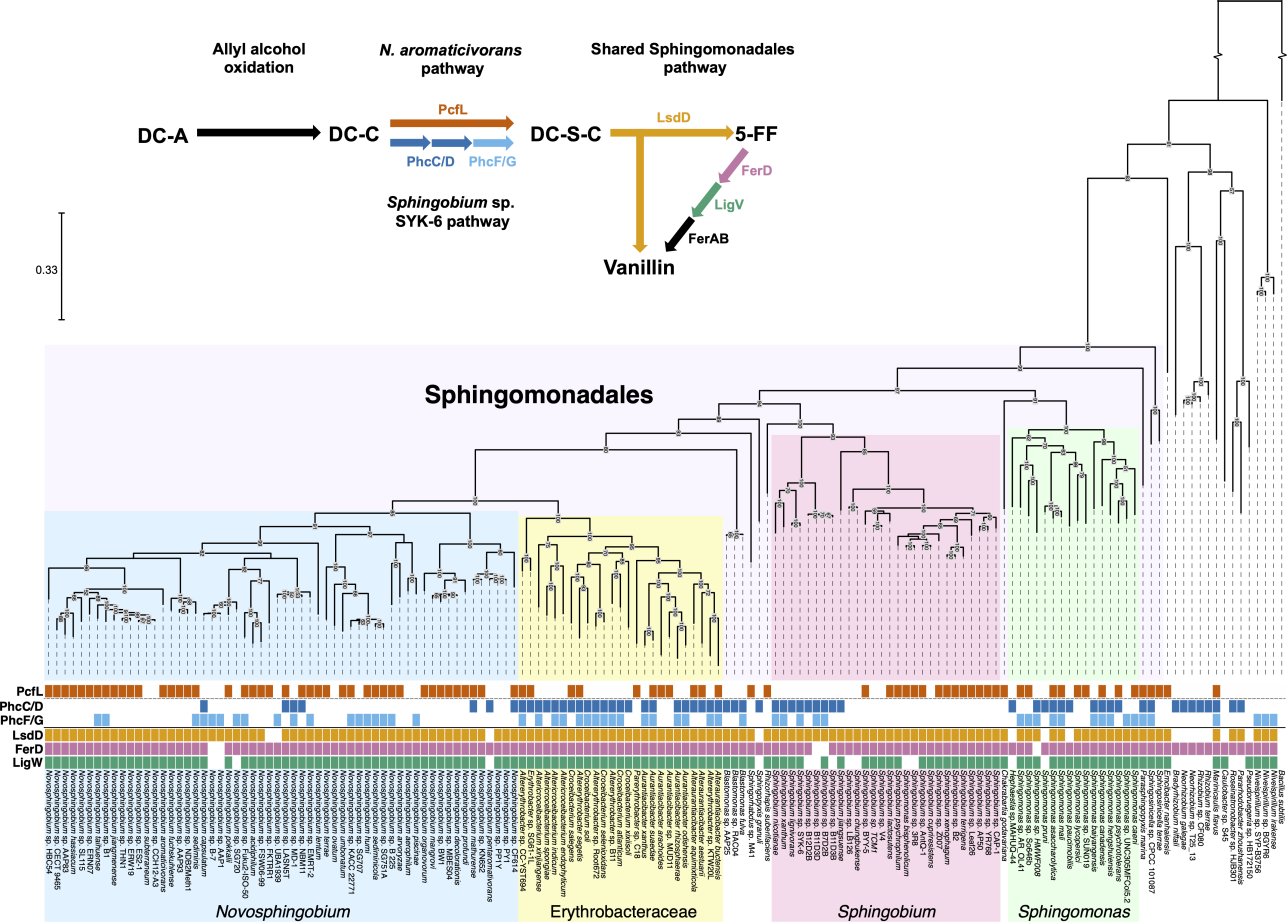
Order Sphingomonadales contains two pathways for conversion of DC-C to DC-S-C and a conserved pathway for DC-S-C catabolism. Phylogeny constructed based on the bacterial reference genes of Alphaproteobacteria containing homologs (>50% amino acid identity, >70% query coverage) of at least two enzymes found in the β-5 linked aromatic catabolic pathways characterized in *N. aromaticivorans* or *Sphingobium* sp. SYK-6. Homologs found in each species are marked by colored boxes. Clades are labeled and color-coded. The scale bar indicates the number of nucleotide substitutions per sequence site. The gap in the outgroup corresponds to 1.5 on the scale bar. A simplified diagram of the DC-A catabolic pathways in *N. aromaticivorans* and *Sphingobium* sp. SYK-6 is shown.

We also used comparative genomics to analyze the distribution of the β-5 linked aromatic catabolic pathways found in *N. aromaticivorans* and *Sphingobium* sp. SYK-6 (Fig. 10). For this analysis, we included the two pairs of enantiospecific enzymes (PhcC/PhcD and PhcF/PhcG) from the *Sphingobium* sp. SYK-6 pathway that are not shared by *N. aromaticivorans*. We found that most species predicted to have the enzymes needed for β-5 linked aromatic catabolism contain homologs of LsdD, FerD, and LigW, but they differ in whether they are predicted to convert DC-C to DC-S-C using a PcfL homolog (*N. aromaticivorans* pathway) or through oxidation and decarboxylation of DC-C (*Sphingobium* sp. SYK-6 pathway). Most of the organisms identified by our search contain homologs of either PcfL or PhcC/PhcD and/or PhcF/PhcG, but 10 species contain homologs of all of these enzymes, suggesting they can convert a phenylcoumaran to a stilbene via both of these pathways.

The largest clades of Alphaproteobacteria with predicted β-5 catabolism capabilities are members of the genera *Novosphingobium*, *Sphingobium*, and *Sphingomonas*, and other members of the family Erythrobacteraceae aside from *Novosphingobium*. Our analysis predicts that the PcfL-dependent formaldehyde-releasing pathway found in *N. aromaticivorans* is common in the genus *Novosphingobium*, while the phenylcoumaran oxidation and decarboxylation pathway discovered in *Sphingobium* sp. SYK-6 is common in other Erythrobacteraceae. The *Sphingobium* clade can be split into two groups, one of which is predicted to use each pathway. By contrast, the *Sphingomonas* clade is comprised of organisms predicted to contain either or both pathways for β-5 linked aromatic catabolism. In total, while the PcfL-dependent pathway is found in 82 Alphaproteobacteria, homologs of both PhcC/PhcD and PhcF/PhcG are found in 32 organisms. Overall, this analysis has revealed a conserved core pathway among the Sphingomonadales for the metabolism of a β-5 linked stilbene and a pair of diverging pathways for the conversion of a phenylcoumaran to a stilbene.

In sum, we identified a catabolic pathway for β-5 linked aromatics in *N. aromaticivorans* that uses four conserved enzymes in addition to several partially redundant enzymes to funnel each monomeric unit into the *N. aromaticivorans* central aromatic pathway. Notably, this work showed that *N. aromaticivorans* uses a heretofore undescribed γ-formaldehyde lyase, PcfL, for converting phenylcoumarans to stilbenes. Future studies should focus on biochemically and mechanistically characterizing PcfL, as well as comparing it to its homolog, LdpA (24, 37), which is reported to generate a stilbene from a β-1 linked aromatic dimer.

The results of this analysis have expanded our knowledge of the aromatic metabolism of *N. aromaticivorans* and the order Sphingomonadales, laying the groundwork for future metabolic engineering to optimize the production of commodity chemicals from additional major components of deconstructed lignin. This *N. aromaticivorans* pathway holds promise for industrial applications since catabolism of β-5 linked aromatics to vanillic acid and ferulic acid requires a minimal set of five gene products, as we demonstrated *in vitro*. These five genes could confer β-5 linked aromatic catabolism on other industrially relevant species. To increase the impact of our findings, future work is needed to assess whether β-5 linked aromatics that have been subjected to different pretreatment conditions are catabolized by *N. aromaticivorans* through a similar pathway to the one elucidated in this study.

## MATERIALS AND METHODS

### Chemicals

Other than those noted below, all chemicals used were analytical grade and were purchased commercially.

(*E)−4-(3-(hydroxymethyl)−5-(3-hydroxyprop-1-en-1-yl)−7-methoxy-2,3-dihydrobenzofuran-2-yl)−2-methoxyphenol* (DC-A) was synthesized in 65% yield by DIBAL-H reduction of 8–5-coupled diferulate (DFA) (45), which was synthesized from ethyl ferulate through peroxidase-H_2_O_2_ oxidative coupling reaction (46). (*E)−3-(2-(4-hydroxy-3-methoxyphenyl)−3-(hydroxymethyl)−7-methoxy-2,3-dihydrobenzofuran-5-yl)acrylaldehyde* (DC-L) was synthesized in 80% yield from DC-A by *p*-benzoquinone oxidation as previously described (47). (*E)−3-(4-hydroxy-3-((E)−4-hydroxy-3-methoxystyryl)−5-methoxyphenyl)acrylic acid* (DC-S-C) was synthesized in 23% yield from DFA by alkali hydrolysis at 90°C as previously described (48). To synthesize (*E)−3-(2-(4-hydroxy-3-methoxyphenyl)−3-(hydroxymethyl)−7-methoxy-2,3-dihydrobenzofuran-5-yl)acrylic acid* (DC-C), DFA was selectively reduced in 95% ethanol by NaBH_4_ to produce the alcohol DFA-1 (32% yield). Protection of phenolic hydroxyl in DFA-1 by phenacyl ether was accomplished in 90% yield. Alkali hydrolysis of the ester group in DFA-2 was performed in 1N NaOH/ethanol (1/1, vol/vol) solution, producing the acid DFA-3 in 85% yield. Finally, deprotection of the phenacyl ether in DFA-3 by Zinc dust in acetic acid resulted in DC-C in 70% yield. The synthesis of DC-A, DC-L, DC-C, and DC-S-C is depicted in Fig. S12A . Each product was confirmed by NMR (Fig. S12B through E ; Table S4).

(*E)−3-(3-formyl-4-hydroxy-5-methoxyphenyl)acrylic acid* (5-FF) was synthesized in 38% yield from ferulic acid by ortho formylation with paraformaldehyde and ammonium acetate in acetic acid as previously described (49). To synthesize (*E)−5-(2-carboxyvinyl)−2-hydroxy-3-methoxybenzoic acid* (5-CF), the phenolic hydroxyl of 5-FF was protected by acetylation in acetic anhydride/pyridine (1/1, vol/vol) to produce acetylated 5-FF. The aldehyde group was then converted to carboxylic acid in 85% yield by Oxone oxidation in DMF as previously described (50). Finally, the acetylated 5-CF was transferred in 95% yield to 5-CF by hydrolysis of the acetate with K_2_CO_3_ in 60% aqueous ethanol. The synthesis of 5-FF and 5-CF is depicted in Fig. S13A. Each product was confirmed by NMR (Fig. S13B and C; Table S4 ).

To generate DC-T-C, DC-S-C was incubated under abiotic conditions in SMB minimal medium supplemented with 1 g/L glucose at 30°C for 2 weeks. DMSO was then added to a 30% final concentration (vol/vol). The resulting product was recovered by ethyl acetate extraction of the SMB buffer solution. After removing the solvent, the crude residue was directly examined by NMR. It was found that DC-S-C was completely converted and the majority of products were two stereoisomers of the 8–8-coupled dimer DC-T-C, which was identified by comparison of their NMR data with those published (Fig. S5A; Table S4 ) (51). This material was used as a 1 mM DC-T-C standard. All other standards were created by dissolving the appropriate compound in DMSO at a final concentration of 100 mM.

### Bacterial strains and growth media

*N. aromaticivorans* strain 12444Δ1879 is referred to as the wild type elsewhere in this paper. In 12444Δ1879, a putative *sacB* homolog (Saro_1879) has been deleted (23) to allow for genomic modifications to be made using the pK18mobsacB plasmid system (52). The 12444PDC strain harbors several gene deletions that allow it to funnel aromatics into the production of the aromatic metabolic pathway intermediate PDC (10). 12444PDC was used as a parent strain for the construction of the deletion mutants used to study DC-A catabolism. All *N. aromaticivorans* strains (Table S5 ) were grown at 30°C and shaken at 200 rpm in SMB minimal medium supplemented with 1 g/L glucose, except where noted. SMB minimal medium was prepared as previously described (23).

*E. coli* NEB5α (New England Biolabs, Ipswich, MA) was used as a plasmid host. *E. coli* WM6026 (53) was used as a conjugal donor for mobilizing plasmids into *N. aromaticivorans* while *E. coli* B834 (54) was used to express recombinant proteins. All *E. coli* strains (Table S5) were grown in lysogeny broth (LB) at 37°C and shaken at 200 rpm, except where noted below.

### RNA-Seq analysis

Four isolated *N. aromaticivorans* PDC12444 colonies were cultured and grown overnight. The next day, the overnight cultures were diluted 1:1 with SMB minimal medium supplemented with 1 g/L glucose and grown for 1 hour. The cultures were then diluted 1:100 into separate cultures of SMB minimal medium supplemented with 1 g/L glucose, 1 g/L glucose plus 0.5 mM DC-A, 1 g/L glucose plus 0.5 mM vanillin, or 1 g/L glucose plus 0.5 mM ferulic acid. These cultures were grown until they reached mid-exponential growth phase, at which point growth was stopped by the 1:8 addition of ice-cold 5% acid phenol:chloroform (5:1) in ethanol. The cells were pelleted by centrifugation (4,300 × *g* for 10 minutes) at 4°C and stored at −80°C. RNA was extracted using hot acid phenol:chloroform (5:1), as previously described (55). RNA was purified using the RNeasy Kit (Qiagen, Germantown, MD), checked for purity by NanoDrop spectrophotometry (OD 260:280 ratio >2.0, OD 260:230 ratio >2.0), visualized after electrophoresis on a 1% agarose gel, and quantified with a Qubit fluorometer.

RNA-Seq library preparation and sequencing were performed by the Joint Genome Institute (JGI) using default parameters. rRNA in the samples was depleted using the QIAseq FastSelect kit (Qiagen, Germantown, MD). Libraries were constructed using the TruSeq stranded mRNA kit (Illumina, San Diego, CA) following standard JGI protocols. The libraries were sequenced on an Illumina NovaSeq to produce 2 × 150 reads. All paired-end FASTQ files were processed through the same pipeline. Reads were trimmed using Trimmomatic version 0.3 with the default settings except for a HEADCROP of 5, LEADING of 3, TRAILING of 3, SLIDINGWINDOW of 3:30, and MINLEN of 36 (56). After trimming, the reads were aligned to the *N. aromaticivorans* DSM12444 genome sequence (GenBank accession GCF_000013325.1) using bwa-mem (version 0.7.17-h5bf99c6_8) with default settings (57). Alignment files were further processed with Picard-tools (version 2.26.10) (https://broadinstitute.github.io/picard/) (*CleanSAM* and *AddOrReplaceReadGroups* commands) and samtools (version 1.2) (*sort* and *index* commands) (58). Paired aligned reads were mapped to gene locations using HTSeq version 0.6.0 (59). The R package edgeR (version 3.30.3) (60) with default settings was used to identify significantly differentially expressed genes from pairwise analyses, using Benjamini and Hochberg false discovery rate (FDR) less than 0.05 as a significance threshold (61). Raw sequencing reads were normalized using the fragments per kilobase per million mapped reads method (FPKM). Fold change, FPKM, and FDR for all genes can be found in File S1.

### Screening a genome-scale RB-TnSeq library

A previously generated RB-TnSeq library in wild-type *N. aromaticivorans* was used to screen for fitness (21). An aliquot of the library was thawed and cultured in LB supplemented with 50 mg/L kanamycin and grown overnight. The culture was diluted 1:100 into three flasks containing 2 g/L glucose in SMB minimal medium and grown to saturation (~6.5 doublings). Each culture was then diluted to a starting cell density of 40 Klett units in SMB minimal medium with 1 g/L glucose or 1 g/L DC-A as the sole carbon source. The cultures were grown to saturation (~6.5 doublings), split into 0.6 mL aliquots, frozen, and stored at −80°C. The cells were harvested by centrifugation (2,300 × *g* for 5 minutes) at 4°C, resuspended in lysis buffer (0.16 mM EDTA and 2% SDS), and incubated at 65°C for 5 minutes. Genomic DNA was extracted using 25:24:1 phenol:chloroform:isoamyl alcohol. Barcode DNA sequences were amplified from the genome using custom indexing primers BarSeq_P1 and BarSeq_P2_IT001 to BarSeq_P2_IT009 (62). Barcode amplicons were quantified using a Qubit fluorometer and pooled before being sequenced at Azenta/GENEWIZ on an Illumina MiSeq with paired-end 150 bp reads (Illumina, San Diego, CA). Barcode frequencies and fitness values were calculated as previously described (62) and can be found for all genes in File S2.

### Heterologous protein expression

To express recombinant proteins, a single isolated colony of each *E. coli* B834 expression strain (see Supplementary Methods for expression strain construction) was cultured in LB medium containing kanamycin (50 mg/L). The next day, the overnight cultures were diluted 1:1 in LB medium and grown for 1 hour at 37°C. Next, flasks containing either 48 mL 2xYPTG medium (16 g/L tryptone, 10 g/L yeast extract, 5 g/L NaCl, 7 g/L KH_2_PO_4_, 3 g/L K_2_HPO_4_, 18 g/L glucose) or 49.5 mL ZMS-80155 auto-inducing medium (63) were inoculated with 2 mL or 0.5 mL of *E. coli* B834 culture, respectively. The 2xYPTG cultures were allowed to grow until their OD600 reached 0.6–0.8, at which point expression of the recombinant protein was induced via the addition of 1 mM isopropyl β-D-1-thiogalactopyranosid (IPTG). Since significant recombinant FdhA was present in inclusion bodies, we added 0.5 M sorbitol and 0.2 M arginine to its culture at the same time we added IPTG (64). The 2xYPTG and ZMS-801555 cultures were both grown overnight at room temperature (~24 hours). The cultures were washed twice with cold S30 buffer supplemented with 2 mM dithiothreitol (DTT) (65) and the cells were harvested by centrifugation (3,000 × *g* for 10 minutes) at 4°C. The cell pellets were flash-frozen in a dry ice-ethanol bath and stored at −80°C. Heterologous expression of His-tagged proteins for purification was performed as described above except the cultures contained 990 mL ZMS-80155 auto-inducing medium and were inoculated with 10 mL *E. coli* B834 culture.

### Harvesting cell extracts

Harvested *E. coli* B834 cells containing the recombinant proteins were resuspended in 12 mL ice-cold S30 buffer supplemented with 2 mM DTT for untagged constructs or in 2.5 mL/g pellet lysis buffer (50 mM NaH_2_PO_4_*H_2_O, 0.5 mM tris(2-carboxyethyl)phosphine, 5 mM imidazole, 100 mM NaCl, 10% glycerol, and 1% Triton-X-100, pH 8.0) for His-tagged constructs. Cells were sonicated on ice using a QSonic sonicator set to amplitude 40 with 20 seconds on and 40 seconds off cycles for 15 minutes. The sonicated solutions were then centrifuged (7,600 × *g* for 20 minutes) at 4°C and the supernatant was collected as a crude cell extract, flash-frozen in a dry ice-ethanol bath, and stored at −80°C.

### Growth experiments

All *N. aromaticivorans* strains (see Supplementary Methods for mutant strain construction) were cultured in triplicate from three isolated colonies and grown overnight. The next day, the cultures were diluted 1:1 in SMB minimal medium supplemented with 1 g/L glucose and incubated for 1 hour before being diluted with additional 1 g/L glucose in SMB minimal medium to the same cell density. A portion of these cultures were centrifuged (2,300 × *g* for 5 minutes), the supernatant was discarded, and the cell pellets were diluted in the appropriate growth medium (SMB minimal medium with 1 g/L glucose and with or without 0.5 mM DC-A). One mL aliquots of the resuspended cells were used to inoculate triplicate flasks containing 19 mL of the appropriate medium, giving a starting cell density of 20–25 Klett units. The cultures were grown for 18 hours and growth was monitored using a Klett-Summerson colorimeter (Fig. S14). At indicated time points, 0.8 mL of the cultures was removed, the cells were pelleted by centrifugation (2,300 × *g* for 5 minutes) at 4°C, and the supernatants were passed through a 0.22-µm PVDF syringe filter to collect extracellular samples that were stored at −80°C for subsequent analysis.

Since DC-A has low solubility in SMB minimal medium, 100 mM DC-A stock in DMSO was added to SMB minimal medium that was heated to 65°C to achieve final concentrations of ~0.45 mM DC-A and 0.5% DMSO after filtering the medium.

### Analysis of extracellular aromatic metabolites

The aromatics in extracellular samples were analyzed on a Shimadzu triple quadrupole liquid chromatography-mass spectrometer (Nexera XR HPLC-8045 MS/MS). The mobile phase was a binary gradient with solvent A (0.2% formic acid in water) and solvent B (methanol) using the protocol in Fig. S15 and flowing at a rate of 0.4 mL/min. The stationary phase was a Phemonenex Kinetex F5 column (2.6 µm pore size, 2.1 mm ID, 150 mm length, P/N: H18-105937). The m/z of peaks was determined using a negative ion mode scan. Aromatic compound standards were generated as described above and used to confirm the identity of unknown chemicals through multiple-reaction monitoring (MRM).

A series of two-fold dilutions were performed to create a standard curve of eight concentrations of each compound. The standard curves were then used to quantify extracellular concentrations of aromatics via MRM (Table S2). The percent yields of individual compounds were calculated using equation (1).


(1) $$\text{percent yield}=\frac{({\text{[aromatic]}}_{\text{final}}\times \text{n)}}{{(\left[\text{DC-A}\right]}_{\text{initial}}\times 2)}\times 100$$

where n = number of aromatic rings in the compound.

### In vitro enzyme activity assays

Crude cell extracts containing individual recombinant proteins were prepared as described above. The cell extracts expressing candidate DC-A catabolism proteins and control *E. coli* B834 cell extract or control extract alone were added to three separate reaction mixtures containing S30 buffer (pH 8.2) supplemented with aromatic substrate and NAD^+^, where appropriate. In candidate test conditions, candidate protein and control extracts each comprised 15% of the final volume and the aromatic substrate and NAD^+^ (where appropriate) concentrations were 0.25 mM and 1 mM, respectively. For the *in vitro* reconstruction of the DC-A catabolic pathway experiment, each of the five protein expression cell extracts made up 5% of the final reaction volume instead. For control reactions, the crude extract from *E. coli* B834 comprised 30% of the final mixture. These reactions were incubated at 30°C for 6 hours and then diluted 1:1 with 40% acetonitrile, 40% methanol, and 100 mM formic acid in water to terminate enzyme activity. The samples were centrifuged (21,000 × *g* for 5 minutes) at 4°C and the supernatants were passed through a 0.22-µm PVDF syringe filter and stored at −80°C for further analysis. Experiments testing *in vitro* activity of purified PcfL and FerD (see Supplementary Methods for protein purification) were performed in the same fashion, except HEPES buffer (pH 7.66) was used in place of S30 buffer and control experiments were conducted by adding additional HEPES buffer instead of crude *E. coli* B834 cell extract.

Analysis of the *in vitro* reaction products was performed on a Shimadzu triple quadrupole liquid chromatography-mass spectrometer as described above. LC traces were collected and reaction products were identified using MRM methods developed from synthetic standards (Table S2).

To assay the relative rate of conversion of substrates to products by candidate ADHs and ALDHs, absorbance at 370 nm was used for measuring DC-L concentration since DC-L absorbs at this wavelength while DC-A and DC-C do not (Fig. S7). *E. coli* B834 cell extracts expressing candidate ADHs or ALDHs as well as control extracts were collected as described above and protein concentration was determined by Bradford assay. The extracts were then diluted with S30 buffer plus 2 mM DTT to a total protein concentration of 2 mg/mL. The dehydrogenase and control *E. coli* B834 cell extracts were each added to triplicate wells of a 96-well plate containing S30 buffer (pH 8.2) supplemented with 0.15 mM DC-A or 0.15 mM DC-L, as well as 1 mM electron acceptor (NAD^+^ or PQQ, where appropriate). The diluted extracts comprised 5% of the final reaction volume. Each enzyme was tested for activity in assays with and without added electron acceptor. After the addition of cell extract to the wells, the 96-well plate was immediately placed in a Tecan Infinite M1000 Pro plate reader set to maintain a temperature of 30°C. At indicated timepoints over the course of 1 hour, the absorbance of DC-L was measured at 370 nm. Control experiments show that NADH does not accumulate significantly in this cell extract system, potentially due to the activity of native *E. coli* dehydrogenases (Fig. S6B). A series of standards created by two-fold dilutions of DC-L in S30 buffer plus 2 mM DTT were used to generate an 8-point standard curve and quantify the concentration of DC-L in the reactions based on absorbance at 370 nm.

Due to the absorbance of PQQ at 370 nm, the activity assay for the putative PQQ-dependent ALDH Saro_2870 was performed as described above except 15 µL samples were collected from the reaction at each indicated time point and diluted 1:1 with 40% acetonitrile, 40% methanol, and 100 mM formic acid in water to terminate enzyme activity. These samples were then diluted 5:1 with S30 buffer and analyzed by LC-MS as described above.

Formaldehyde was measured as a product of PcfL activity using small aliquots of the purified protein reaction mixtures and the Invitrogen Formaldehyde Fluorescent Detection Kit (Invitrogen, Carlsbad, CA). To test for conversion of NAD^+^ to NADH by FerD, assays were performed as described above for both the purified FerD and FerD-containing cell extract, except the S30 or HEPES buffer was supplemented with 0.4 mM NAD^+^ and 0.4 mM 5-FF. NAD^+^ and NADH were quantified using small aliquots of the reactions and the Sigma Aldrich NAD/NADH Quantitation Kit (Sigma Aldrich, St. Louis, MO).

#### Phylogenetic analysis

Predicted homologs of DC-A catabolism genes were identified using NCBI protein-protein BLAST to search all genomes in the NCBI database as of July 2023, excluding uncultured/environmental sample sequences and using cut-offs of 50% amino acid identity and 70% query coverage. All bacteria containing homologs of at least two *N. aromaticivorans* DC-A catabolism enzymes (PcfL, FerD, LigW, and LsdD) were used to create a phylogenetic tree. Alphaproteobacteria containing homologs of at least two *N. aromaticivorans* DC-A catabolism enzymes (PcfL, FerD, LigW, and LsdD) and/or *Sphingobium* sp. SYK-6 DC-A catabolism enzymes that differ from *N. aromaticivorans* (PhcC/PhcD and PhcF/PhcG) were used to create an additional phylogenetic tree.

Phylogenetic analysis was performed on genomes identified in these BLAST searches (Table S6) using GDTB-Tk (version 2.1.1, release 207_v2) to identify and align the bacterial reference genes using default parameters (66). The multiple sequence alignment file was used to construct maximum likelihood trees using RAxML-ng (version 0.9.0) using model LG+G8+Fand default parameters (67). *Bacillus subtilis* subsp. subtilis str. 168 was used as an outgroup. Phylogenetic trees were visualized in TreeViewer (version 2.2.0) (68).

## Data Availability

The raw RB-TnSeq reads are available from SRA as BioProject PRJNA1082384. The raw RNA-Seq reads are available from GEO through accession number GSE259365.

## SUPPLEMENTAL MATERIAL

File S1 mbio.01718-24-s0001.xlsx Full RNA-Seq data.

File S2 mbio.01718-24-s0002.xlsx Full RB-TnSeq data.

Supplemental Material mbio.01718-24-s0003.docx Supplemental figures, methods, and tables.

ASM does not own the copyrights to Supplemental Material that may be linked to, or accessed through, an article. The authors have granted ASM a non-exclusive, world-wide license to publish the Supplemental Material files. Please contact the corresponding author directly for reuse.
