# Supplementary material for: Catabolism of β-5 linked aromatics by Novosphingobium aromaticivorans
Source: mBio. 2024 Jul 16;15(8):e01718-24. doi: 10.1128/mbio.01718-24 (PMC11323797; doi:10.1128/mbio.01718-24)
Supplement: Supplemental Material — Supplemental figures, methods, and tables. [file mbio.01718-24-s0003.docx]

Catabolism of β-5 linked aromatics by *Novosphingobium aromaticivorans*

**Authors:** Fletcher Metz,^a,b,c^ Abigail M. Olsen,^a,b^ Fachuang Lu,^a,b^ Kevin S. Myers,^a,b^ Marco N. Allemann,^d^ Joshua K. Michener,^d^ Daniel R. Noguera,^a,b,e^ Timothy J. Donohue^a,b,f,*^

^a^ DOE Great Lakes Bioenergy Research Center, University of Wisconsin, Madison, Wisconsin, USA

^b^ Wisconsin Energy Institute, University of Wisconsin, Madison, Wisconsin, USA

^c^ Laboratory of Genetics, University of Wisconsin, Madison, Wisconsin, USA

^d^ Biosciences Division, Oak Ridge National Laboratory, Oak Ridge, TN, USA

^e^ Department of Civil and Environmental Engineering, University of Wisconsin, Madison, Wisconsin, USA

^f^ Department of Bacteriology, University of Wisconsin, Madison, Wisconsin, USA

^*^ Corresponding author

**Supplementary Figures**

**Supplementary Figure 1. Trace amounts of DC-L transiently accumulate during DC-A catabolism.** DC-L concentration in extracellular medium of 12444PDC grown in SMB minimal medium with DC-A plus glucose as carbon sources. Error bars represent standard deviation across biological triplicates.

**Supplementary Figure 2. Genome-wide screens identify candidate genes for DC-A catabolism.** Dot plot (log_2_ scale) of RNA-Seq (y-axis) and RB-TnSeq (x-axis) data sets, with each dot representing a single gene. The horizontal and vertical red lines mark a 2-fold increase in transcript abundance when *N. aromaticivorans* PDC12444 is grown on DC-A compared to A) glucose or B) ferulic acid and a 2-fold abundance reduction of a disrupted gene when a *N. aromaticivorans* DSM12444 RB-TnSeq library is grown on DC-A compared to glucose, respectively. The five candidate genes investigated in this study are labeled in red.

**Supplementary Figure 3. Formaldehyde is released when PcfL converts DC-C to DC-S-C.** Concentration of formaldehyde after 6 hours of incubating *in vitro* reactions containing DC-C and either purified PcfL or HEPES buffer as a control. Error bars represent standard deviation across triplicates.

**Supplementary Figure 4. FdhA acts on formaldehyde released during DC-A catabolism.** A) Metabolite concentrations in extracellular medium of 12444PDCΔ*fdhA* grown in SMB minimal medium with DC-A plus glucose as carbon sources. B) Formaldehyde concentration in extracellular medium of 12444PDC or 12444PDCΔ*fdhA* grown in SMB minimal medium with DC-A plus glucose as carbon sources. Error bars represent standard deviation across biological triplicates.

**Supplementary Figure 5. DC-S-C abiotically homodimerizes in aqueous solutions to form DC-T-C.** A) ^13^C NMR spectrum of the product obtained when DC-S-C is incubated in SMB minimal medium supplemented with 1 g/L glucose. The structure of the resulting compound, DC-T-C, is shown. B) Loss of DC-S-C over time in various solutions. Note that some DC-S-C visually precipitated in the water condition. Error bars represent standard deviation across triplicates.

**Supplementary Figure 6. FerD is an NAD^+^-dependent aldehyde dehydrogenase.** A) Representative HPLC chromatograms of *in vitro* reactions containing 5-FF and either control *E. coli* B834 cell extract or cell extract of *E. coli* B834 expressing recombinant FerD without added NAD^+^. B) Ratio of NAD^+^ to NADH after 6 hours incubating *in vitro* reactions containing 5-FF and NAD^+^ along with purified FerD, cell extract of *E. coli* B834 expressing recombinant FerD, or control *E. coli* B834 cell extract. Error bars represent standard deviation across triplicates.

**Supplementary Figure 7. Differences in DC-A, DC-L, and DC-C absorbance can be leveraged in colorimetric assays.** UV-Vis traces of 0.2 mM solutions of DC-A, DC-L, and DC-C in S30 buffer.

**Supplementary Figure 8. FerD converts vanillin to vanillic acid.** Representative HPLC chromatograms of *in vitro* reactions containing vanillin and either control *E. coli* B834 cell extract or cell extract of *E. coli* B834 expressing recombinant FerD.

**Supplementary Figure 9. PcfL exhibits activity on DC-A and DC-L *in vitro*.** Representative HPLC chromatograms of *in vitro* reactions containing A) DC-A or B) DC-L and either control *E. coli* B834 cell extract or cell extract of *E. coli* B834 expressing recombinant PcfL. C) Structures of proposed stilbene compounds based on m/z of the *in vitro* reaction products.

**Supplementary Figure 10.** **Proposed *N. aromaticivorans* catabolic pathway for DC-A, accounting for the ability of PcfL to act on DC-A, DC-L, and DC-C.** The allylic alcohol is oxidized to an aldehyde and then to a carboxylic acid by dehydrogenases. The five-member ring of DC-C is opened by PcfL to form DC-S-C, which is then cleaved by LsdD into vanillin and 5-FF. 5-FF is oxidized to 5-CF by FerD and other dehydrogenases before it is decarboxylated by LigW to form ferulic acid. Metabolism of ferulic acid and vanillin to PDC by *N. aromaticivorans* has been previously described (1, 2). The gene products involved in metabolism of formaldehyde following oxidation by FdhA represent a hypothetical pathway based on homology with known S-glutathione hydrolases (Saro_2822) (3) and the subunits of a formate dehydrogenase complex (Saro_0732, Saro_0733, and Saro_0735) (4). Steps that differ from those proposed in Fig. 4 are marked with blue arrows.

**Supplementary Figure 11. The full *N. aromaticivorans* DC-A catabolic pathway is exclusive to Alphaproteobacteria.** Phylogeny constructed based on the bacterial reference genes of bacteria containing homologs (>50% amino acid identity, >70% query coverage) of at least two enzymes found in the *N. aromaticivorans* β-5 linked aromatic pathway. The bacterial species are sorted by class. The colored bars to the right of the tree indicate the proportion of each class containing a homolog of each enzyme. The scale bar indicates the number of nucleotide substitutions per sequence site. A simplified diagram of the DC-A catabolic pathway in *N. aromaticivorans* is shown.

**Supplementary Figure 12. DC-A, DC-L, DC-C, and DC-S-C synthesis.** A) Synthetic routes to DC-A, DC-L, DC-C, and DC-S-C. B-E) ^13^C NMR (acetone-d_6_) spectra and structures of synthetic B) DC-A, C) DC-L, D) DC-C, and E) DC-S-C.

**Supplementary Figure 13. DC-S-C and DC-T-C synthesis.** A) Synthetic routes to 5-FF and 5-CF. B-C) ^13^C NMR (acetone-d_6_) spectra and structures of synthetic B) 5-FF and C) 5-CF.

**Supplementary Figure 14. Growth of 12444PDC and 12444PDC mutant strains**. Growth curves of 12444PDC and 12444PDC mutant strains in SMB minimal medium containing 0.5 mM DC-A and 1g/L glucose as carbon sources. Error bars represent standard deviation across biological triplicates.

**Supplementary Figure 15. Solvent B (MeOH) percent protocol for HPLC method.** Trace of percent solvent B over time. Solvent A was 0.2% formic acid in water.

**Supplementary Figure 16.** **Differences in DC-S-C and DC-T-C can be leveraged in colorimetric assays.** UV-Vis traces of 0.2 mM solutions of DC-S-C and DC-T-C in S30 buffer.

**Supplementary Methods**

*Construction of in-frame deletion mutants*

Gene deletion mutants were constructed using 12444PDC as a parent strain and the pK18mobsacB suicide plasmid. This plasmid was linearized via polymerase chain reaction (PCR) as previously described (5). Regions of *N. aromaticivorans* genomic DNA ~1,000 bp upstream and downstream of each gene of interest (Supplementary Table 7) were amplified via PCR using the primers listed in Supplementary Table 8 that contain overhanging regions complementary to the ends of linearized pK18mobsacB. NEBuilder HiFi Assembly system (New England Biolabs, Ipswich, MA) was used to insert the amplified fragments into the linearized plasmid, creating a construct in which the genomic regions upstream and downstream of the gene to be deleted are adjacent to each other with no coding region between them. All plasmids used are listed in Supplementary Table 9.

These plasmids were transformed into *E. coli* NEB5α by heat shock. Plasmids were isolated from NEB5α cultures using the QIAprep Miniprep Kit (Qiagen, Germantown, MD) and the insert regions of the plasmids were amplified and submitted for Sanger sequencing at Functional Biosciences (Madison, WI) or the University of Wisconsin–Madison DNA Sequencing core facility. Once the sequences of these plasmids were verified, they were transformed via heat shock into *E. coli* WM6026, which served as a conjugal donor to mobilize the plasmids into *N. aromaticivorans* as previously described (6), except that the SMB minimal medium contained 1 g/L glucose.

*Construction of protein expression strains*

Plasmids for recombinant protein expression were constructed using pVP302K, which was linearized via PCR using the primers listed in Supplementary Table 10. Codon optimized (Benchling Biological Software) gBlocks (Supplementary Table 11) of genes of interest (Supplementary Table 7) for heterologous recombinant protein expression were obtained from Integrated DNA Technologies (San Diego, California) and amplified by PCR using the primers in Supplementary Table 9 that contain overhanging regions complementary to the ends of linearized pVP302K. NEBuilder HiFi Assembly system was used to insert the amplified gBlocks into the linearized plasmid, yielding untagged expression plasmids for all genes as well as N-terminal His-tagged constructs with a TEV-protease cleavage site between the tag and the protein for PcfL and FerD. All plasmids used are listed in Supplementary Table 9.

These pVP302K derivatives were transformed into *E. coli* NEB5α and their sequences were verified as described above. They were then transformed into *E. coli* B834 by heat shock.

*Protein purification*

PcfL and FerD were purified from the crude cell extract by fast protein liquid chromatography. The crude cell extracts were applied directly to a Ni-NTA column and washed with buffer A (50 mM NaH_2_PO_4_*H_2_O, 0.5 mM tris(2-carboxyethyl)phosphine, 25 mM imidazole, and 200 mM NaCl, pH 7.5). The His-tagged proteins bound to the resin were eluted with Buffer B (50 mM NaH_2_PO_4_*H_2_O, 0.5 mM tris(2-carboxyethyl)phosphine, 500 mM imidazole, and 300 mM NaCl, pH 7.5). The eluted proteins were collected and concentrated in Buffer C (50 mM NaH_2_PO_4_ * H_2_O, 0.5 mM tris(2-carboxyethyl)phosphine, 10 mM imidazole, and 100 mM NaCl, pH 7.5) using a 10 kDA MWCO centrifugal filter and hanging basket centrifugation (3,000 x g) at 4 °C. Protein concentration was quantified by Bradford protein assay measuring absorbance at 595 nm and the purified proteins were diluted to ~2 mg/mL protein by addition of buffer C. They were then treated overnight at 4 °C with 1 mg TEV-protease per ~30 mg of protein. The protease-treated samples were applied to a Ni-NTA column and the proteins were eluted with buffer C and the high imidazole buffer B was used afterwards to elute any remaining protein. A 10 kDA MWCO centrifugal filter and hanging basket centrifugation (3,000 x g) at 4 °C was used to concentrate the proteins, wash them twice with HEPES buffer (50 mM HEPES, 20 mM NaCl, pH 7.5), and concentrate them again. Fractions were saved throughout the purification process and protein content in each fraction was analyzed by sodium dodecyl sulfate polyacrylamide gel electrophoresis. Glycerol was added to the purified, concentrated proteins to a final concentration of 20% before they were flash frozen in a dry ice-ethanol bath and stored at -80 °C. A Bradford protein assay measuring absorbance at 595 nm was used to determine the final protein concentration.

*Analysis of extracellular formaldehyde*

Extracellular medium samples were collected as described in the Materials and Methods and analyzed for extracellular formaldehyde by the Great Lakes Bioenergy Research Center Metabolomics Lab. Formaldehyde concentrations were measured by headspace analysis using an Agilent 7890 Gas Chromatogram equipped with a LECO Pegasus BT time-of-flight mass spectrometry and controlled using LECO’s ChromTOF software v4.72.0.0. The samples were prepared in 20 mL headspace vials (Restek, Cat#23082) by diluting 100 µL of filtered medium into 5 mL of water containing p-TSA as the internal standard. The diluted samples were loaded onto a L-PAL 3 auto-sampler equipped with a 2.5 mL headspace syringe (PAL system, Cat#PAL3-Sys-008655). Prior to injection, each sample was transferred to an agitator preheated to 70 °C and incubated for 40 minutes at 350 rpm prior to loading 500 µL of the headspace gas into the syringe. The sample was injected into a 120 °C inlet with a 50:1 split ratio onto a Stabilwax-DA column (Restek, 30 m × 0.25 mm × 0.5 µm, Cat#11038) with helium as the mobile phase flowing at a constant 1 mL/min. The temperature program was set at 40 °C for 4.20 minutes, followed by a 40 °C/minute ramp up to 200 °C. The transfer line to the MS was set to 210 °C. The MS source was set to 200 °C and had an acquisition delay of 135 seconds. The chromatogram data was collected from 135–55 seconds at 10 spectra/sec covering the mass range of 10–350 *m/z*. Quantification was performed using p-TSA as the internal standard with a 10-point calibration curve.

*DC-S-C abiotic dimerization assay*

The time-dependent abiotic conversion of DC-S-C to DC-T-C was measured in water, DMSO, S30 buffer, and SMB minimal medium supplemented with 1 g/L glucose in a 96-well plate. DC-S-C was added in triplicate to each medium to a concentration of 0.2 mM and the 96-well plate was immediately placed in a Tecan Infinite M1000 reader set to maintain a temperature of 30 °C. Every hour for 18 hours, absorbance of DC-S-C was measured at 370 nm since DC-S-C absorbs at 370 nm while DC-T-C does not (Fig. S16). A series of 2-fold dilutions were performed to create a standard curve of eight concentrations of DC-S-C and of DC-T-C in each medium. The standard curves were then used to quantify extracellular concentrations of these aromatics based on absorbance at 370 nm.

*Absorbance spectra of standards*

To identify the wavelengths at which to measure absorbance in the ADH and ALDH *in vitro* assays and DC-S-C abiotic dimerization assay, the absorbance of standards was determined with the goal of identifying wavelengths at which either solely a substrate or solely a product absorbs. Triplicate 0.2 mM mixtures of DC-A, DC-L, and DC-C in S30 buffer and 0.2 mM standards of DC-S-C and DC-T-C in SMB minimal medium supplemented with 1g/L glucose were created and their absorbance was measured from 230 nm to 500 nm in a Tecan Infinite M1000 reader.

**Supplementary Tables**

**Supplementary Table 1.** HPLC-MS multiple reaction monitoring conditions and elution times for the compounds analyzed in this study.

| **Compound** | **MW (g/mol)** | **Parent Ion (-) m/z** | **Transition 1 m/z** | **Transition 2 m/z** | **Transition 3 m/z** | **Elution Time (min)^1^** |
| --- | --- | --- | --- | --- | --- | --- |
| PDC | 184.10 | 183.30 | 111.00 | 139.05 | 95.00 | 1.11 |
| Vanillic Acid | 168.14 | 167.25 | 152.05 | 108.05 | 123.05 | 2.13 |
| Vanillin | 152.15 | 151.15 | 136.00 | 92.00 | 108.00 | 2.41 |
| Ferulic Acid | 194.18 | 193.25 | 134.15 | 178.00 | 149.10 | 2.99 |
| 5-carboxyferulate | 238.19 | 237.10 | 134.10 | 178.10 | 149.15 | 3.36 |
| 5-formylferulate | 222.19 | 221.10 | 206.10 | 134.10 | 162.10 | 3.87 |
| DC-A | 358.38 | 357.15 | 203.10 | 339.15 | 221.20 | 5.25 |
| DC-C | 372.37 | 371.15 | 352.30 | 341.20 | 191.05 | 5.62 |
| DC-L | 356.37 | 355.15 | 337.15 | 219.05 | 190.05 | 5.97 |
| DC-S-C | 342.34 | 341.15 | 267.15 | 326.15 | 282.10 | 6.72 |
| DC-T-C | 682.68 | 681.25 | 339.20 | 637.25 | 324.15 | 6.84 |

^1^ Elution times can differ when measurements are taken on different days. The elution times listed are those that are found in the HPLC chromatograms shown in this study.

**Supplementary Table 2.** DC-A catabolism candidate genes identified from RNA-Seq and RB-TnSeq data.

| **Name** | **Locus Tag** | **Transcript Increase^1^** | **Abundance Reduction^2^** | **Annotation** | **Function in DC-A Catabolism** |
| --- | --- | --- | --- | --- | --- |
| *pcfL* | Saro_0796 | 5.39 | -5.71 | Nuclear transport factor 2 family protein | Phenylcoumaran ring opening |
| *fdhA* | Saro_0874 | 2.17 | -3.27 | S-(hydroxymethyl) glutathione dehydrogenase | Formaldehyde metabolism;  Allylic alcohol oxidation |
| *lsdD* | Saro_0802 | 3.80 | -5.34 | Carotenoid oxygenase family protein | Stilbene cleavage |
| *ferD* | Saro_0797 | 4.25 | -4.18 | NAD^+^-dependent succinate-semialdehyde dehydrogenase | 5-FF oxidation;  Allylic aldehyde oxidation |
| *ligW* | Saro_0799 | 4.65 | -1.90 | Amidohydrolase | 5-CF decarboxylation |

^1^ log_2_ comparing transcript abundance when *N. aromaticivorans* PDC12444 is grown on DC-A plus glucose compared and vanillin plus glucose.

^2^ log_2_ comparing abundance of *N. aromaticivorans* DSM12444 transposon mutants grown on DC-A to those grown on glucose.

**Supplementary Table 3.** Candidate ADHs and ALDHs identified from RNA-Seq data.

| **Name/ Locus Tag** | **Enzyme Class** | **Percent of Total ADH or ALDH Transcripts^1^** | **Activity on DC-A or DC-L** |
| --- | --- | --- | --- |
| FdhA | ADH | 46.65% | Yes |
| Saro_0995 | ADH | 2.16% | Yes |
| Saro_1431 | ADH | 2.95% | No |
| Saro_1476 | ADH | 2.38% | No |
| Saro_2795 | ADH | 2.17% | No |
| Saro_2870 | ADH | 30.89% | No |
| Saro_3899 | ADH | 3.41% | Yes |
| Saro_3463 | ADH | 3.84% | No |
| Saro_0060 | ALDH | 2.36% | No |
| FerD | ALDH | 7.43% | Yes |
| Saro_1104 | ALDH | 16.02% | Yes |
| Saro_1197 | ALDH | 12.16% | Yes |
| Saro_1410 | ALDH | 10.16% | No |
| LigV | ALDH | 2.04% | No |
| Saro_1967 | ALDH | 22.20% | No |
| Saro_2869 | ALDH | 14.74% | Yes |
| Saro_3848 | ALDH | 4.76% | No |

^1^ Percent of total putative ADH or ALDH transcripts when *N. aromaticivorans* 12444PDC is grown in the presence of DC-A.

**Supplementary Table 4.** ^1^H and ^13^C NMR (acetone-d_6_) analysis of indicated compounds.

| **Compound** | **^1^H NMR Data** | **^13^C NMR Data** |
| --- | --- | --- |
| DC-A | 3.52, 3.78-3.88, 3.81,3.85, 4.19, 5.56, 6.23, 6.52, 6.80, 6.87, 6.94, 6.97, 7.03 | 54.70, 56.13, 56.21, 63.33, 64.49, 88.45, 110.30, 111.41, 115.58, 115.96, 119.51, 128.28, 130.29, 130.42, 131.82, 134.28, 145.09, 147.19, 148.28, 148.82 |
| DC-L | 3.61, 3.82, 3.91, 3.87-3.91, 5.65, 6.65, 6.81, 6.88, 7.04, 7.29, 7.32, 7.59, 9.63 | 54.25, 56.29, 56.46, 64.32, 89.39, 110.59, 113.56, 115.76, 119.64, 119.73, 127.14, 129.00, 131.24, 133.75, 145.65, 147.55, 148.46, 152.41, 154.10, 193.77 |
| DC-C | 3.59 (m, 1H), 3.82 (s, 3H, -OMe), 3.83-3.92 (m, 2H), 3.90 (s, 3H, -OMe), 4.18, 5.63, 6.38 (d, J= 15.92 Hz), 6.81 (d, J= 8.15 Hz), 6.88 (dd, J= 8.15, 1.93 Hz), 7.05 (d, J= 1.93 Hz), 7.23 (br-s), 7.25 (br-s), 7.61(d, J= 15.92 Hz) | 54.36, 56.20, 56.33, 64.28, 89.14, 110.45, 113.12, 115.67, 116.00, 118.73, 119.67, 129.01, 130.88, 133.86, 145.46, 145.98, 147.41, 148.38, 151.54, 168.04. |
| DC-S-C | 3.91 (s, OMe), 3.95 (s, OMe), 6.44 (d, J= 15.9 Hz),6.83(d, J= 8.1 Hz), 7.05 (dd, J= 8.1, 2.0, ), 7.22 (d, J= 2.0 Hz), 7.23 (d, J= 1.9 Hz), 7.31 and 7.33 (ABqt, ∆ν_AB_=7.39Hz, J_AB_ = 16.5 Hz), 7.54 (d, J=1.9 Hz), 7.63 (1 H, d, J=15.9Hz) | 56.10, 56.44, 108.96, 109.89, 115.90, 116.18, 120.41, 120.82, 121.10, 125.33, 126.83, 130.57, 130.77, 146.21, 146.88, 147.46, 148.49, 148.71, 168.35 |
| 5-FF | 3.98 (s, 3H, OMe), 6.52 (d, J= 16.0 Hz), 7.64 (d, J =16.0 Hz), 7.64 and 7.64 (ABqt, ∆ν_AB_=3.56 Hz, J_AB_ = 2.15 Hz), 10.15 (s, -CHO) | 56.68, 116.36, 118.06, 122.11, 125.31, 127.39, 144.34, 149.74, 154.02, 167.70, 196.04 (-CHO) |
| 5-CF | 3.95 (s, OMe), 6.48 (d, J= 15.95 Hz), 7.59 (d, J= 2.0 Hz), 7.62 (d, J= 15.95 Hz), 7.71 (d, J= 2.0 Hz) | 56.50 (OMe), 113.17, 115.43, 117.60, 123.87, 126.30, 144.75, 150.12, 155.52, 167.78, 172.64 |
| DC-T-C (*threo* isomer) | 3.62(s), 3.98 (s), 4.13 (d, J= 3.64 Hz), 5.53 (d, J= 3.64 Hz), 6.30 (d, J=1.90 Hz), 6.39 (d, J=15.90 Hz), 6.53 (dd, J = 8.15, 1.90 Hz), 6.67 (d, J = 8.15 Hz), 7.30 (d, J= 1.50 Hz), 7.35 (d, J= 1.50 Hz), 7.59 (d, J= 15.90 Hz) | 55.76, 55.98, 56.48, 87.12, 109.10, 113.15, 115.59, 117.72, 118.56, 118.77, 129.60, 130.13, 133.63, 144.20, 145.65, 146.96, 148.30, 151.41, 169.60 |
| DC-T-C (*meso* isomer) | 3.78 (s, OMe), 3.91 (s, OMe), 4.18 (d, J= 6.15 Hz), 5.52 (d, J= 6.15 Hz), 6.25 (d, J =15.90 Hz), 6.80 (d, J= 1.2 Hz), 6.82 (d, J= 8.10 Hz), 6.84 (dd, J= 8.10, 1.36 Hz), 6.98 (d, J=1.56 Hz), 7.30 (d, J= 1.56 Hz), 7.52 (d, J= 15.90 Hz) | 53.50 (C-8), 56.22, 56.38, 88.67, 110.83, 113.57, 115.85, 116.43, 118.48, 120.12, 129.35, 130.11, 132.91, 145.65, 145.70, 147.81, 148.50, 151.92, 167.93 |

**Supplementary Table 5.** Bacterial strains used in this study.

| **Strain** | **Relevant Characteristics** | **Source** |
| --- | --- | --- |
| 12444Δ1879 | WT *N. aromaticivorans* Δ1879 (*sacB-*) | (5) |
| 12444PDC | 12444Δ1879 Δ2819 (*ligI*) Δ2864 (*desC*) Δ2865 (*desD*) | (2) |
| 12444PDCΔ*pcfL* | 12444PDC Δ0796 (*pcfL*) | This study |
| 12444PDCΔ*ferD* | 12444PDC Δ0797 (*ferD*) | This study |
| 12444PDCΔ*ligW* | 12444PDC Δ0799 (*ligW*) | This study |
| 12444PDCΔ*lsdD* | 12444PDC Δ0802 (*lsdD*) | This study |
| 12444PDCΔ*fdhA* | 12444PDC Δ0874 (*fdhA*) | This study |
| *E. coli* NEB5α | *fhuA2* Δ*(argF-lacZ)U169 phoA glnV44* Φ*80* Δ*(lacZ)M15 gyrA96 recA1 relA1 endA1 thi-1 hsdR17* | New England Biolabs |
| *E. coli* WM6026 | lacI*^q^*, rrnB3, ΔlacZ4787, hsdR514, ΔaraBAD567, ΔrhaBAD568, rph-1, attλ::pAE12(ΔoriR6K-cat::Frt5), ΔendA::Frt, uidA(ΔMluI)::pir, attHK::pJK1006D(oriR6K-cat::Frt5; trfA::Frt) dap | (7) |
| *E. coli* B834 | F^-^ *hsdS metE gal ompT* | (8) |

**Supplementary Table 6.** Organisms included in the phylogenetic analyses in Fig. 10 and Fig. S11.

| **Scientific Name** | **Assembly Accession Number** | **Class** | **Analysis in this Study** |
| --- | --- | --- | --- |
| *Alteraurantiacibacter aestuarii* | GCF_009827405.1 | Alphaproteobacteria | Fig. 10; Fig. S11 |
| *Alteraurantiacibacter aquimixticola* | GCF_004965515.1 | Alphaproteobacteria | Fig. 10; Fig. S11 |
| *Alteraurantiacibacter buctensis* | GCF_009827655.1 | Alphaproteobacteria | Fig. 10; Fig. S11 |
| *Altererythrobacter segetis* | GCF_011320115.1 | Alphaproteobacteria | Fig. 10; Fig. S11 |
| *Altererythrobacter* sp. B11 | GCF_003569745.1 | Alphaproteobacteria | Fig. 10; Fig. S11 |
| *Altererythrobacter* sp. CC-YST694 | GCF_020539485.1 | Alphaproteobacteria | Fig. 10; Fig. S11 |
| *Altererythrobacter* sp. KTW20L | GCF_023501975.1 | Alphaproteobacteria | Fig. 10; Fig. S11 |
| *Altererythrobacter* sp. Root672 | GCF_001427865.1 | Alphaproteobacteria | Fig. 10; Fig. S11 |
| *Altericroceibacterium endophyticum* | GCF_009827595.1 | Alphaproteobacteria | Fig. 10; Fig. S11 |
| *Altericroceibacterium indicum* | GCF_009828105.1 | Alphaproteobacteria | Fig. 10; Fig. S11 |
| *Altericroceibacterium spongiae* | GCF_003610805.1 | Alphaproteobacteria | Fig. 10; Fig. S11 |
| *Altericroceibacterium xinjiangense* | GCF_003958635.1 | Alphaproteobacteria | Fig. 10; Fig. S11 |
| *Aurantiacibacter arachoides* | GCF_009827335.1 | Alphaproteobacteria | Fig. 10; Fig. S11 |
| *Aurantiacibacter odishensis* | GCF_003605195.1 | Alphaproteobacteria | Fig. 10; Fig. S11 |
| *Aurantiacibacter rhizosphaerae* | GCF_009807005.1 | Alphaproteobacteria | Fig. 10; Fig. S11 |
| *Aurantiacibacter* sp. MUD11 | GCF_026967575.1 | Alphaproteobacteria | Fig. 10; Fig. S11 |
| *Aurantiacibacter suaedae* | GCF_005434915.1 | Alphaproteobacteria | Fig. 10; Fig. S11 |
| *Aurantiacibacter xanthus* | GCF_003584015.1 | Alphaproteobacteria | Fig. 10; Fig. S11 |
| *Blastomonas fulva* | GCF_003431825.1 | Alphaproteobacteria | Fig. 10; Fig. S11 |
| *Blastomonas* sp. AAP25 | GCF_001295965.1 | Alphaproteobacteria | Fig. 10; Fig. S11 |
| *Blastomonas* sp. RAC04 | GCF_001713435.1 | Alphaproteobacteria | Fig. 10; Fig. S11 |
| *Bradyrhizobium niftali* | GCF_004571025.1 | Alphaproteobacteria | Fig. 10 |
| *Caulobacter* sp. S45 | GCF_009765965.1 | Alphaproteobacteria | Fig. 10; Fig. S11 |
| *Chakrabartia godavariana* | GCA_023260075.1 | Alphaproteobacteria | Fig. 10; Fig. S11 |
| *Croceibacterium atlanticum* | GCF_001008165.2 | Alphaproteobacteria | Fig. 10; Fig. S11 |
| *Croceibacterium salegens* | GCF_009827435.1 | Alphaproteobacteria | Fig. 10; Fig. S11 |
| *Croceibacterium selenioxidans* | GCF_018599195.1 | Alphaproteobacteria | Fig. 10; Fig. S11 |
| *Croceibacterium soli* | GCF_009828065.1 | Alphaproteobacteria | Fig. 10; Fig. S11 |
| *Croceibacterium xixiisoli* | GCF_009827305.1 | Alphaproteobacteria | Fig. 10; Fig. S11 |
| *Emcibacter nanhaiensis* | GCF_006385175.1 | Alphaproteobacteria | Fig. 10; Fig. S11 |
| *Erythrobacter* sp. SG61-1L | GCF_001305965.1 | Alphaproteobacteria | Fig. 10; Fig. S11 |
| *Hephaestia* sp. MAHUQ-44 | GCF_023806085.1 | Alphaproteobacteria | Fig. 10 |
| *Marinicaulis flavus* | GCF_002943565.1 | Alphaproteobacteria | Fig. 10; Fig. S11 |
| *Neorhizobium galegae* | GCF_008806425.1 | Alphaproteobacteria | Fig. 10 |
| *Neorhizobium* sp. T25_13 | GCF_002968675.1 | Alphaproteobacteria | Fig. 10 |
| *Niveispirillum irakense* | GCF_000429645.1 | Alphaproteobacteria | Fig. 10; Fig. S11 |
| *Niveispirillum* sp. BGYR6 | GCF_027568365.1 | Alphaproteobacteria | Fig. 10; Fig. S11 |
| *Niveispirillum* sp. SYP-B3756 | GCF_009495745.1 | Alphaproteobacteria | Fig. 10; Fig. S11 |
| *Novosphingobium acidiphilum* | GCF_000429005.1 | Alphaproteobacteria | Fig. 10; Fig. S11 |
| *Novosphingobium aerophilum* | GCF_014230345.1 | Alphaproteobacteria | Fig. 10; Fig. S11 |
| *Novosphingobium aromaticivorans* | GCF_900102455.1 | Alphaproteobacteria | Fig. 10; Fig. S11 |
| *Novosphingobium arvoryzae* | GCF_014652615.1 | Alphaproteobacteria | Fig. 10; Fig. S11 |
| *Novosphingobium capsulatum* | GCF_031454595.1 | Alphaproteobacteria | Fig. 10; Fig. S11 |
| *Novosphingobium decolorationis* | GCF_018417475.1 | Alphaproteobacteria | Fig. 10; Fig. S11 |
| *Novosphingobium fuchskuhlense* | GCF_001519075.1 | Alphaproteobacteria | Fig. 10; Fig. S11 |
| *Novosphingobium hassiacum* | GCF_014196055.1 | Alphaproteobacteria | Fig. 10; Fig. S11 |
| *Novosphingobium humi* | GCF_028607105.1 | Alphaproteobacteria | Fig. 10; Fig. S11 |
| *Novosphingobium jiangmenense* | GCF_015694345.1 | Alphaproteobacteria | Fig. 10; Fig. S11 |
| *Novosphingobium lentum* | GCF_001590965.1 | Alphaproteobacteria | Fig. 10; Fig. S11 |
| *Novosphingobium mangrovi* | GCF_022818885.1 | Alphaproteobacteria | Fig. 10; Fig. S11 |
| *Novosphingobium mathurense* | GCF_900168325.1 | Alphaproteobacteria | Fig. 10; Fig. S11 |
| *Novosphingobium organovorum* | GCF_022832435.1 | Alphaproteobacteria | Fig. 10; Fig. S11 |
| *Novosphingobium ovatum* | GCF_009909235.1 | Alphaproteobacteria | Fig. 10; Fig. S11 |
| *Novosphingobium pentaromativorans* | GCA_003241455.1 | Alphaproteobacteria | Fig. 10 |
| *Novosphingobium piscinae* | GCF_014230355.1 | Alphaproteobacteria | Fig. 10; Fig. S11 |
| *Novosphingobium pokkalii* | GCF_014652855.1 | Alphaproteobacteria | Fig. 10; Fig. S11 |
| *Novosphingobium profundi* | GCF_018491765.1 | Alphaproteobacteria | Fig. 10; Fig. S11 |
| *Novosphingobium sediminicola* | GCF_014196525.1 | Alphaproteobacteria | Fig. 10; Fig. S11 |
| *Novosphingobium sediminis* | GCF_007991615.1 | Alphaproteobacteria | Fig. 10; Fig. S11 |
| *Novosphingobium* sp. AAP1 | GCF_001295765.1 | Alphaproteobacteria | Fig. 10 |
| *Novosphingobium* sp. AAP83 | GCF_001295795.1 | Alphaproteobacteria | Fig. 10; Fig. S11 |
| *Novosphingobium* sp. AAP93 | GCF_001296055.1 | Alphaproteobacteria | Fig. 10; Fig. S11 |
| *Novosphingobium* sp. B 225 | GCF_002198665.1 | Alphaproteobacteria | Fig. 10; Fig. S11 |
| *Novosphingobium* sp. B-7 | GCF_000410615.1 | Alphaproteobacteria | Fig. 10 |
| *Novosphingobium* sp. B1 | GCF_900176395.1 | Alphaproteobacteria | Fig. 10; Fig. S11 |
| *Novosphingobium* sp. BW1 | GCF_008107685.1 | Alphaproteobacteria | Fig. 10; Fig. S11 |
| *Novosphingobium* sp. CCH12-A3 | GCF_001556015.1 | Alphaproteobacteria | Fig. 10; Fig. S11 |
| *Novosphingobium* sp. CECT 9465 | GCF_920987055.1 | Alphaproteobacteria | Fig. 10; Fig. S11 |
| *Novosphingobium* sp. CF614 | GCF_900113255.1 | Alphaproteobacteria | Fig. 10; Fig. S11 |
| *Novosphingobium* sp. EMRT-2 | GCF_005145025.1 | Alphaproteobacteria | Fig. 10; Fig. S11 |
| *Novosphingobium* sp. ERN07 | GCF_012641335.1 | Alphaproteobacteria | Fig. 10; Fig. S11 |
| *Novosphingobium* sp. ERW19 | GCF_012641315.1 | Alphaproteobacteria | Fig. 10; Fig. S11 |
| *Novosphingobium* sp. ES2-1 | GCF_015169775.1 | Alphaproteobacteria | Fig. 10; Fig. S11 |
| *Novosphingobium* sp. FKTRR1 | GCF_020404405.1 | Alphaproteobacteria | Fig. 10; Fig. S11 |
| *Novosphingobium* sp. FSW06-99 | GCF_001519065.1 | Alphaproteobacteria | Fig. 10; Fig. S11 |
| *Novosphingobium* sp. Fuku2-ISO-50 | GCF_001519055.1 | Alphaproteobacteria | Fig. 10; Fig. S11 |
| *Novosphingobium* sp. HBC54 | GCF_029436685.1 | Alphaproteobacteria | Fig. 10; Fig. S11 |
| *Novosphingobium* sp. KACC 22771 | GCF_028736195.1 | Alphaproteobacteria | Fig. 10; Fig. S11 |
| *Novosphingobium* sp. KN65.2 | GCF_001368935.1 | Alphaproteobacteria | Fig. 10; Fig. S11 |
| *Novosphingobium* sp. LASN5T | GCF_003856955.1 | Alphaproteobacteria | Fig. 10; Fig. S11 |
| *Novosphingobium* sp. MBES04 | GCF_000813185.1 | Alphaproteobacteria | Fig. 10; Fig. S11 |
| *Novosphingobium* sp. MD-1 | GCF_001014975.1 | Alphaproteobacteria | Fig. 10; Fig. S11 |
| *Novosphingobium* sp. NBM11 | GCF_015390225.1 | Alphaproteobacteria | Fig. 10; Fig. S11 |
| *Novosphingobium* sp. NDB2Meth1 | GCF_900117425.1 | Alphaproteobacteria | Fig. 10; Fig. S11 |
| *Novosphingobium* sp. PP1Y | GCF_000253255.1 | Alphaproteobacteria | Fig. 10; Fig. S11 |
| *Novosphingobium* sp. PY1 | GCF_017312445.1 | Alphaproteobacteria | Fig. 10; Fig. S11 |
| *Novosphingobium* sp. SG707 | GCF_012275515.1 | Alphaproteobacteria | Fig. 10; Fig. S11 |
| *Novosphingobium* sp. SG720 | GCF_012275365.1 | Alphaproteobacteria | Fig. 10; Fig. S11 |
| *Novosphingobium* sp. SG751A | GCF_013149295.1 | Alphaproteobacteria | Fig. 10; Fig. S11 |
| *Novosphingobium* sp. SL115 | GCF_026672515.1 | Alphaproteobacteria | Fig. 10; Fig. S11 |
| *Novosphingobium* sp. THN1 | GCF_003454795.1 | Alphaproteobacteria | Fig. 10; Fig. S11 |
| *Novosphingobium* sp. UBA1939 | GCF_002336885.1 | Alphaproteobacteria | Fig. 10; Fig. S11 |
| *Novosphingobium subterraneum* | GCF_000807925.1 | Alphaproteobacteria | Fig. 10; Fig. S11 |
| *Novosphingobium taihuense* | GCF_007830315.1 | Alphaproteobacteria | Fig. 10; Fig. S11 |
| *Novosphingobium terrae* | GCF_017163935.1 | Alphaproteobacteria | Fig. 10 |
| *Novosphingobium umbonatum* | GCF_004005905.1 | Alphaproteobacteria | Fig. 10; Fig. S11 |
| *Pararhodobacter zhoushanensis* | GCF_003990445.1 | Alphaproteobacteria | Fig. 10; Fig. S11 |
| *Parasphingopyxis marina* | GCF_014237875.1 | Alphaproteobacteria | Fig. 10; Fig. S11 |
| *Parerythrobacter* sp. C18 | GCF_030140925.1 | Alphaproteobacteria | Fig. 10; Fig. S11 |
| *Pseudoruegeria* sp. HB172150 | GCF_013184805.1 | Alphaproteobacteria | Fig. 10; Fig. S11 |
| *Rhizobium* sp. CF080 | GCF_000282095.2 | Alphaproteobacteria | Fig. 10 |
| *Rhizobium terrae* | GCF_003425685.1 | Alphaproteobacteria | Fig. 10; Fig. S11 |
| *Rhizorhapis suberifaciens* | GCF_014200045.1 | Alphaproteobacteria | Fig. 10; Fig. S11 |
| *Roseinatronobacter* sp. HJB301 | GCF_028745735.1 | Alphaproteobacteria | Fig. 10; Fig. S11 |
| *Sphingobium chungbukense* | GCF_001005725.1 | Alphaproteobacteria | Fig. 10; Fig. S11 |
| *Sphingobium cupriresistens* | GCF_004152865.1 | Alphaproteobacteria | Fig. 10; Fig. S11 |
| *Sphingobium jiangsuense* | GCF_014196495.1 | Alphaproteobacteria | Fig. 10; Fig. S11 |
| *Sphingobium lactosutens* | GCF_013393185.1 | Alphaproteobacteria | Fig. 10; Fig. S11 |
| *Sphingobium lignivorans* | GCF_014203955.1 | Alphaproteobacteria | Fig. 10; Fig. S11 |
| *Sphingobium nicotianae* | GCF_018603885.1 | Alphaproteobacteria | Fig. 10; Fig. S11 |
| *Sphingobium psychrophilum* | GCF_012927105.1 | Alphaproteobacteria | Fig. 10; Fig. S11 |
| *Sphingobium* sp. 3R8 | GCF_020166615.1 | Alphaproteobacteria | Fig. 10; Fig. S11 |
| *Sphingobium* sp. AntQ-1 | GCF_028538045.1 | Alphaproteobacteria | Fig. 10; Fig. S11 |
| *Sphingobium* sp. AP50 | GCF_900109095.1 | Alphaproteobacteria | Fig. 10; Fig. S11 |
| *Sphingobium* sp. B11D3B | GCF_025961735.1 | Alphaproteobacteria | Fig. 10; Fig. S11 |
| *Sphingobium* sp. B11D3D | GCF_025961755.1 | Alphaproteobacteria | Fig. 10 |
| *Sphingobium* sp. B12D2B | GCF_025961775.1 | Alphaproteobacteria | Fig. 10; Fig. S11 |
| *Sphingobium* sp. B2 | GCF_007693735.1 | Alphaproteobacteria | Fig. 10; Fig. S11 |
| *Sphingobium* sp. B7D2B | GCF_025961895.1 | Alphaproteobacteria | Fig. 10; Fig. S11 |
| *Sphingobium* sp. BYY-5 | GCF_022758885.1 | Alphaproteobacteria | Fig. 10; Fig. S11 |
| *Sphingobium* sp. CAP-1 | GCF_009720145.1 | Alphaproteobacteria | Fig. 10; Fig. S11 |
| *Sphingobium* sp. LB126 | GCF_002795205.1 | Alphaproteobacteria | Fig. 10; Fig. S11 |
| *Sphingobium* sp. Leaf26 | GCF_001421665.1 | Alphaproteobacteria | Fig. 10; Fig. S11 |
| *Sphingobium* sp. SYK-6 | GCF_000283515.1 | Alphaproteobacteria | Fig. 10; Fig. S11 |
| *Sphingobium* sp. TCM1 | GCF_001650725.1 | Alphaproteobacteria | Fig. 10; Fig. S11 |
| *Sphingobium* sp. V4 | GCF_029590555.1 | Alphaproteobacteria | Fig. 10; Fig. S11 |
| *Sphingobium* sp. YR768 | GCF_900111125.1 | Alphaproteobacteria | Fig. 10; Fig. S11 |
| *Sphingobium* sp. Z007 | GCF_900013445.1 | Alphaproteobacteria | Fig. 10; Fig. S11 |
| *Sphingobium terrigena* | GCF_003591655.1 | Alphaproteobacteria | Fig. 10; Fig. S11 |
| *Sphingobium xanthum* | GCF_019737615.1 | Alphaproteobacteria | Fig. 10; Fig. S11 |
| *Sphingobium xenophagum* | GCF_002288285.1 | Alphaproteobacteria | Fig. 10; Fig. S11 |
| *Sphingomonas asaccharolytica* | GCF_001598355.1 | Alphaproteobacteria | Fig. 10; Fig. S11 |
| *Sphingomonas baiyangensis* | GCF_005144715.1 | Alphaproteobacteria | Fig. 10; Fig. S11 |
| *Sphingomonas bisphenolicum* | GCF_024349785.1 | Alphaproteobacteria | Fig. 10; Fig. S11 |
| *Sphingomonas caeni* | GCF_026013415.1 | Alphaproteobacteria | Fig. 10; Fig. S11 |
| *Sphingomonas canadensis* | GCF_026013525.1 | Alphaproteobacteria | Fig. 10; Fig. S11 |
| *Sphingomonas hengshuiensis* | GCF_000935025.1 | Alphaproteobacteria | Fig. 10; Fig. S11 |
| *Sphingomonas lycopersici* | GCF_026130585.1 | Alphaproteobacteria | Fig. 10; Fig. S11 |
| *Sphingomonas mali* | GCF_001598415.1 | Alphaproteobacteria | Fig. 10; Fig. S11 |
| *Sphingomonas paucimobilis* | GCF_001029575.1 | Alphaproteobacteria | Fig. 10; Fig. S11 |
| *Sphingomonas pruni* | GCF_001598455.1 | Alphaproteobacteria | Fig. 10; Fig. S11 |
| *Sphingomonas psychrotolerans* | GCF_002796605.1 | Alphaproteobacteria | Fig. 10; Fig. S11 |
| *Sphingomonas* sp. AR_OL41 | GCF_029911635.1 | Alphaproteobacteria | Fig. 10; Fig. S11 |
| *Sphingomonas* sp. HMWF008 | GCA_003061185.1 | Alphaproteobacteria | Fig. 10; Fig. S11 |
| *Sphingomonas* sp. So64.6b | GCF_014171475.1 | Alphaproteobacteria | Fig. 10; Fig. S11 |
| *Sphingomona*s sp. SUN019 | GCF_024758705.1 | Alphaproteobacteria | Fig. 10; Fig. S11 |
| *Sphingomonas* sp. UNC305MFCol5.2 | GCF_000712135.1 | Alphaproteobacteria | Fig. 10; Fig. S11 |
| *Sphingopyxis granuli* | GCF_001956775.1 | Alphaproteobacteria | Fig. 10 |
| *Sphingorhabdus* sp. M41 | GCF_001586275.1 | Alphaproteobacteria | Fig. 10; Fig. S11 |
| *Sphingosinicella* sp. CPCC 101087 | GCF_004151485.1 | Alphaproteobacteria | Fig. 10; Fig. S11 |
| *Sphingosinicella terrae* | GCF_003347635.1 | Alphaproteobacteria | Fig. 10; Fig. S11 |
| *Caldimonas tepidiphila* | GCF_003569765.1 | Betaproteobacteria | Fig. S11 |
| *Glaciimonas soli* | GCF_009497155.1 | Betaproteobacteria | Fig. S11 |
| *Massilia cavernae* | GCF_003590855.1 | Betaproteobacteria | Fig. S11 |
| *Noviherbaspirillum humi* | GCF_900188095.1 | Betaproteobacteria | Fig. S11 |
| *Luteimonas* sp. BDR2-5 | GCF_021191695.1 | Gammaproteobacteria | Fig. S11 |
| *Pseudomonas capeferrum* | GCF_000731675.1 | Gammaproteobacteria | Fig. S11 |
| *Pseudomonas* sp. LS1212 | GCF_024741815.1 | Gammaproteobacteria | Fig. S11 |
| *Pseudomonas* sp. R5(2019) | GCF_009905435.1 | Gammaproteobacteria | Fig. S11 |
| *Geodermatophilus sabuli* | GCF_900215145.1 | Actinomycetes | Fig. S11 |
| *Lipingzhangella halophila* | GCF_014203805.1 | Actinomycetes | Fig. S11 |
| *Pseudonocardia* sp. CNS-004 | GCF_001942185.1 | Actinomycetes | Fig. S11 |
| *Pseudonocardia* sp. DSM 110487 | GCF_019468565.1 | Actinomycetes | Fig. S11 |
| *Pseudonocardia hierapolitana* | GCF_007994075.1 | Actinomycetes | Fig. S11 |
| *Rhodococcus jostii* | GCF_900105375.1 | Actinomycetes | Fig. S11 |
| *Rhodococcus opacus* | GCF_019856255.1 | Actinomycetes | Fig. S11 |
| *Streptomyces* sp. NRRL S-813 | GCF_000718945.1 | Actinomycetes | Fig. S11 |
| *Streptomyces spiralis* | GCF_014654675.1 | Actinomycetes | Fig. S11 |
| *Thermopolyspora flexuosa* | GCF_006716785.1 | Actinomycetes | Fig. S11 |
| *Bacillus subtilis* subsp. subtilis str. 168 | GCF_000155325.1 | Bacilli | Fig. 10; Fig. S11 |
| *Paenibacillus* sp. tmac-D7 | GCF_006519665.1 | Bacilli | Fig. S11 |

**Supplementary Table 7.** *N. aromaticivorans* genes analyzed in this study and their associated locus tags. Unnamed alcohol dehydrogenase gene products (ADHs) and aldehyde dehydrogenase gene products (ALDHs) investigated are labeled by enzyme class.

| ***N. aromaticivorans* gene** | **Saro_ Locus Tag** | **SARO_RS Locus Tag** |
| --- | --- | --- |
| PcfL | Saro_0796 | SARO_RS03975 |
| FerD | Saro_0797 | SARO_RS03980 |
| LigW | Saro_0799 | SARO_RS03990 |
| LsdD | Saro_0802 | SARO_RS04005 |
| FdhA | Saro_0874 | SARO_RS04375 |
| LigV | Saro_1668 | SARO_RS08360 |
| Putative ADH | Saro_0995 | SARO_RS04970 |
| Putative ADH | Saro_1431 | SARO_RS07175 |
| Putative ADH | Saro_1476 | SARO_RS07405 |
| Putative ADH | Saro_2795 | SARO_RS14810 |
| Putative ADH | Saro_2870 | SARO_RS14555 |
| Putative ADH | Saro_3463 | SARO_RS18190 |
| Putative ADH | Saro_3899 | SARO_RS17300 |
| Putative ALDH | Saro_0060 | SARO_RS02990 |
| Putative ALDH | Saro_1104 | SARO_RS05510 |
| Putative ALDH | Saro_1197 | SARO_RS05980 |
| Putative ALDH | Saro_1410 | SARO_RS07070 |
| Putative ALDH | Saro_1967 | SARO_RS09870 |
| Putative ALDH | Saro_2869 | SARO_RS14550 |
| Putative ALDH | Saro_3848 | SARO_RS17045 |

**Supplementary Table 8.** Primers used to create gene deletion mutants. Capitalized regions are complementary to the end of linearized pK18mobsacB. Red regions are complementary to each other, as are blue regions. Underlined bases do not match template.

| **PCR Reaction** | **Primers** |
| --- | --- |
| Linearize pK18mobsacB | pK18msB AseI ampl F:  ctgtcgtgccagctgcattaatg  pK18msB –MCS XbaI R:  gaacatctagaaagccagtccgcagaaac |
| Amplify region upstream of *pcfL* | PcfL pk18 F:  CGATTCATTAATGCAGCTGGCACGACAGcttttcgcttctccagctcgg  PcfL Del R.2:  cccacccgcaatctcttatttccggtccaactcccatcaatttagtttgtc |
| Amplify region downstream of *pcfL* | PcfL pk18 R.2:  GTTTCTGCGGACTGGCTTTCTAGATGTTCcttccacgatgaagcgggttgg  PcfL Del F.2:  gacaaactaaattgatgggagttggaccggaaataagagattgcgggtggg |
| Amplify region upstream of *ferD* | FerD pk18 F:  CGATTCATTAATGCAGCTGGCACGACAGcggctcgcgcaatttgttagtaag  FerD Del R.3:  ctgccgaccgacaccgcaattatatttaatctccggaagccttttgcctg |
| Amplify region downstream of *ferD* | FerD pk18 R.2:  GTTTCTGCGGACTGGCTTTCTAGATGTTCcggatcatgcgcaggtagacgtc  FerD Del F.3:  caggcaaaaggcttccggagattaaatataattgcggtgtcggtcggcag |
| Amplify region upstream of *ligW* | LigW pk18 F:  CGATTCATTAATGCAGCTGGCACGACAGgaaggcgcaatccggagttctcc  LigW Del R:  ccctcccggcgctggtcaaaggcaggcttccttcccgggaag |
| Amplify region downstream of *ligW* | LigW pk18 R:  GTTTCTGCGGACTGGCTTTCTAGATGTTCtccagtggaagccgggagtgacc  LigW Del F:  cttcccgggaaggaagcctgcctttgaccagcgccgggaggg |
| Amplify region upstream of *lsdD* | LsdD pk18 F.4:  CGATTCATTAATGCAGCTGGCACGACAGgggggctaaccgccagtctctatcttc  LsdD Del R.4:  gcaatacatacaatattgcaaggaggatgccgccgcatgatccagcccggag |
| Amplify region downstream of *lsdD* | LsdD pk18 R.3:  GTTTCTGCGGACTGGCTTTCTAGATGTTCccaacaggcagccgaggatag  LsdD Del F.4:  ctccgggctggatcatgcggcggcatcctccttgcaatattgtatgtattgc |
| Amplify region upstream of *fdhA* | FdhA pk18 F:  CGATTCATTAATGCAGCTGGCACGACAGctgacacggatctctcctcaacc  FdhA Del R:  gtaaaccgtgtaaacccgttcaggtattgctacagccctgttaaattgcg |
| Amplify region downstream of *fdhA* | FdhA pk18 R:  cgcaatttaacagggctgtagcaatacctgaacgggtttacacggtttac  FdhA Del F:  cgcaatttaacagggctgtagcaatacctgaacgggtttacacggtttac |

**Supplementary Table 9.** Plasmids used in this study.

| **Plasmid** | **Relevant Characteristics** | **Source** |
| --- | --- | --- |
| pK18mobsacB | pMB1ori *sacB kan*R mobT oriT(RP4) *lacZ*α | (9) |
| pVP302K | lac promoter lacI, Tev site *rtxA* (*V. cholera*) *kan*R; coding sequence for 8×His-tag | (10) |
| pK18mobsacBΔ*pcfL* | pK18mobsacB containing genomic regions flanking *pcfL* | This study |
| pK18mobsacBΔ*lsdD* | pK18mobsacB containing genomic regions flanking *lsdD* | This study |
| pK18mobsacBΔ*ferD* | pK18mobsacB containing genomic regions flanking *ferD* | This study |
| pK18mobsacBΔ*ligW* | pK18mobsacB containing genomic regions flanking *ligW* | This study |
| pK18mobsacBΔ*fdhA* | pK18mobsacB containing genomic regions flanking *fdhA* | This study |
| pVP302K-PcfL | pVP302K containing codon optimized PcfL | This study |
| pVP302K-PcfL-NTag | pVP302K containing codon optimized PcfL downstream of His-tag coding sequence and Tev protease site | This study |
| pVP302K-LsdD | pVP302K containing codon optimized LsdD | This study |
| pVP302K-FerD | pVP302K containing codon optimized FerD | This study |
| pVP302K-FerD-NTag | pVP302K containing codon optimized FerD downstream of His-tag coding sequence and Tev protease site | This study |
| pVP302K-LigW | pVP302K containing codon optimized LigW | This study |
| pVP302K-FdhA | pVP302K containing codon optimized FdhA | This study |
| pVP302K-LigV | pVP302K containing codon optimized LigV | This study |
| pVP302K-0995 | pVP302K containing codon optimized Saro_0995 | This study |
| pVP302K-1431 | pVP302K containing codon optimized Saro_1431 | This study |
| pVP302K-1476 | pVP302K containing codon optimized Saro_1476 | This study |
| pVP302K-2795 | pVP302K containing codon optimized Saro_2795 | This study |
| pVP302K-2870 | pVP302K containing codon optimized Saro_2870 | This study |
| pVP302K-3463 | pVP302K containing codon optimized Saro_3463 | This study |
| pVP302K-3899 | pVP302K containing codon optimized Saro_3899 | This study |
| pVP302K-0060 | pVP302K containing codon optimized Saro_0060 | This study |
| pVP302K-1104 | pVP302K containing codon optimized Saro_1104 | This study |
| pVP302K-1197 | pVP302K containing codon optimized Saro_1197 | This study |
| pVP302K-1410 | pVP302K containing codon optimized Saro_1410 | This study |
| pVP302K-1967 | pVP302K containing codon optimized Saro_1967 | This study |
| pVP302K-2869 | pVP302K containing codon optimized Saro_2869 | This study |
| pVP302K-3848 | pVP302K containing codon optimized Saro_3848 | This study |

**Supplementary Table 10.** Primers used to create recombinant protein expression plasmids. Capitalized DNA sequences are complementary to the end of linearized pVP302K.

| **PCR Reaction** | **Primers** |
| --- | --- |
| Linearize pVP302K with no His-tag | pVP302K No His Lin F:  taacagaaagccgaaaataacaaagttagc  pVP302K No His Lin R:  catggttaatttctcctctttaatgaattctgtg |
| Linearize pVP302K with an N-terminal His-tag | pVP302K N-Term Lin F:  cagaaagccgaaaataacaaagttagcctgag  pVP302K N-Term Lin R:  tgcgatcgcgctctgaaaatacag |
| Amplify PcfL gBlock (no His-tag construct) | pVP302K No His PcfL HiFi F:  TAAAGAGGAGAAATTAACCATGtccgatagcaatcagattgcc  pVP302K No His PcfL HiFi R:  TGTTATTTTCGGCTTTCTGTTAtttccgcgcattttcgc |
| Amplify FerD gBlock (no His-tag construct) | pVP302K No His FerD HiFi F:  TAAAGAGGAGAAATTAACCATGactgcgtacccttctctcc  pVP302K No His FerD HiFi R:  TGTTATTTTCGGCTTTCTGTTAcccttcatgtaccgctttgg |
| Amplify LigW gBlock | pVP302K No His LigW HiFi F:  TAAAGAGGAGAAATTAACCATGacacaagacctgaagaccgg  pVP302K No His LigW HiFi R:  TGTTATTTTCGGCTTTCTGTTAaagtttaaaccatttttcagcgttgg |
| Amplify LsdD gBlock | pVP302K No His LsdD HiFi F:  TAAAGAGGAGAAATTAACCATGgctcaatttccgaataccccaag  pVP302K No His LsdD HiFi R:  TGTTATTTTCGGCTTTCTGTTAtgcggccaggaccttttc |
| Amplify FdhA gBlock | pVP302K No His LsdD HiFi F:  TAAAGAGGAGAAATTAACCATGctaagcgacaggcacgtcaaag  pVP302K No His LsdD HiFi R:  TGTTATTTTCGGCTTTCTGTTAgaacaccactactgaacgaatcgatttac |
| Amplify PcfL gBlock (N-terminal His-tag construct) | pVP302K-N PcfL HiFi F:  AAATCTGTATTTTCAGAGCGCGATCGCAtccgatagcaatcagattgccg  pVP302K-N PcfL HiFi R:  GGCTAACTTTGTTATTTTCGGCTTTCTGttatttccgcgcattttcgcg |
| Amplify FerD gBlock (N-terminal His-tag construct) | pVP302K-N FerD HiFi F:  AAATCTGTATTTTCAGAGCGCGATCGCAactgcgtacccttctctccacatg  pVP302K-N FerD HiFi R:  GGCTAACTTTGTTATTTTCGGCTTTCTGttacccttcatgtaccgctttggtgac |
| Amplify LigV gBlock | Exp LigV F:  CATTAAAGAGGAGAAATTAACCatgcagtttgaacgtatcaatccgatg  Exp LigV R:  GTTTAAACTATTAATGATGATGttaaattggatagtgacctggttggg |
| Amplify Saro_0995 gBlock | 0995 Exp F:  CATTAAAGAGGAGAAATTAACCatgaaagccgccgtactc  0995 Exp R:  GTTTAAACTATTAATGATGATGttattgatcaaacacaataacagaacg |
| Amplify Saro_1431 gBlock | 1431 Exp F:  CATTAAAGAGGAGAAATTAACCatgacaatcaatacaattcgcgtacg  1431 Exp R:  CGTTTAAACTATTAATGATGATttaacaaaaatgacggcagctctg |
| Amplify Saro_1476 gBlock | 1476 Exp F:  CATTAAAGAGGAGAAATTAACCatgttgggacgtgcatcgg  1476 Exp R:  GTTTAAACTATTAATGATGATGttacgtgatcgtcggatcgatc |
| Amplify Saro_2795 gBlock | Exp 2795 F:  CATTAAAGAGGAGAAATTAACCatggcggcaattaatcttccccg  Exp 2795 R:  GTTTAAACTATTAATGATGATGttagccaaagacttcggcatagaggc |
| Amplify Saro_2870 gBlock | Exp 2870x F:  CATTAAAGAGGAGAAATTAACCatgcgattgaaagtactgggacttatgg  Exp 2870 R:  GTTTAAACTATTAATGATGATGttagccacctttggcttctaaag |
| Amplify Saro_3463 gBlock | Exp 3463 F:  CATTAAAGAGGAGAAATTAACCatgattccgcatggtgaacattcaatgctg  Exp 3463 R:  GTTTAAACTATTAATGATGATGttatggcaccaaaaccagagcgccac |
| Amplify Saro_3899 gBlock | Exp 3899 F:  CATTAAAGAGGAGAAATTAACCatggacgcatacgctgcaattatc  Exp 3899 R:  GTTTAAACTATTAATGATGATGttacattttgagaatggcttttatcgcttttc |
| Amplify Saro_0060 gBlock | Exp 0060 F:  CATTAAAGAGGAGAAATTAACCatgtctacacagcctgcaaccatagctg  Exp 0060 R:  GTTTAAACTATTAATGATGATGttatggacgagtttgcccgcttcc |
| Amplify Saro_1104 gBlock | Exp 1104 F:  CATTAAAGAGGAGAAATTAACCatgcgcgaacggctacagcaatacattg  Exp 1104 R:  GTTTAAACTATTAATGATGATGttaggcaggcaggccgctgatcg |
| Amplify Saro_1197 gBlock | Exp 1197 F:  CATTAAAGAGGAGAAATTAACCatgactgcccctaccgcc  Exp 1197 R:  GTTTAAACTATTAATGATGATGttactgctgatgacgatatacagcc |
| Amplify Saro_1410 gBlock | Exp 1410 F:  CATTAAAGAGGAGAAATTAACCatgggttaccgggttgtagtggtg  Exp 1410 R:  CATTAAAGAGGAGAAATTAACCatgcagtttgaacgtatcaatccgatg |
| Amplify Saro_1967 gBlock | Exp 1967 F:  CATTAAAGAGGAGAAATTAACCatggcgatcaaagttgcgataaac  Exp 1967 R:  GTTTAAACTATTAATGATGATGttaaaggaatttcgccattgctcc |
| Amplify Saro_2869 gBlock | Exp 2869 F:  CATTAAAGAGGAGAAATTAACCatgaatgacatgactaccatctc  Exp 2869 R:  GTTTAAACTATTAATGATGATGttacatttgaataattactgttttagtctc |
| Amplify Saro_3848 gBlock | Exp 3848 F:  CATTAAAGAGGAGAAATTAACCatggctacgcagttgagaagtgcag  Exp 3848 R:  GTTTAAACTATTAATGATGATGttactgatcgaacattccggtacgacc |

**Supplementary Table 11.** gBlocks of *N. aromaticivorans* genes codon optimized for *E. coli* and used to create heterologous protein expression constructs.

| **gBlock** | **Sequence** |
| --- | --- |
| PcfL gBlock | ccgatagcaatcagattgccgcgcttgaaagtcgcctgaatgacctcgaaaggcgactgacggttagagaggacgagctggacgtacgcaaactccagcatttatacggttatctgattgataaatgcatgtataacgagacagttgacctgttcacagaagatggggaagtgcggttctttggtggcgtatggaaaggcaaggagggcatccgccgtttgtacgttgaacgttttcagaaacgtttcacctatggcaataacggcccgattgatgggttcctgttagatcatccacaacttcaagatattattcacgtgcaggatgatggggtcacggctttgggccgcgcgcgttccatgatgcaagccggtcgccacaaggattatgagggagatgcacctcatctgaaagcgcgtcagtggtgggaaggtggtatatacgaaaacacttataaaaaagtggatggcgtgtggcgtatgcatatcctaaactacatgccgatctggcacgcagattttgaaagcggctgggccaataccccgcacgaatacgttccttttcccaaagtcacctatccagaagacccgactggaccggatgaactgattgctgaccattggttatggccgacccataagctgaacccctttcacatgaaacatccggtgacgggtgaggaaatggtcgcacagcgctggcagggtgacatcgatcgcgaaaatgcgcggaaataa |
| FerD gBlock | actgcgtacccttctctccacatgattattgacggtgcccgtgtcagcggcggaggacgtcgcacccacgcggtcgtcaatccggctaccggagagaccatcggtgaactgccgctggcagaagttgcagatctggatcgagcgttagaagtagcggcgaagggcttccgtatttggcgtgacagcacaccgcagcagcgcgcagccgtgttacagggcgcggcccggctgatgctggaacggcaagaggatctcgctcgcatagccacgatggaagaaggtaaaaccctgcccgaggcgcgcatcgaagttctgatgaacgtgggcctgttcaatttttacgctggagaagtatttcgtttatatggccgaaccctagtgcgccctgcgggtcagagaagcacgatcacgcatgaaccggtagggccggtggccgcctttgctccgtggaactttccgcttgggaatccaggtcgcaaactgggcgcgccaattgccgccggttgctcggtgattctaaaagcggcggaagaaacgccggcttcagcgttaggggtgctgcaatgtctgctggatgctggcctgcctaaagaagtggcccaggctgtgttcggtgtgcctgacgaggtgagtcgccacctgttgggcagttccgttatccgcaagctctcgtttacaggttctaccgtcatcggcaagcatctgatgcgacttgcagccgacaacatgttgcgtacaactatggagcttggcggccatggtcctgtcttagttttcggtgatgcagatattgacaaagcgctcgataccatggcagcttccaaatatcgtaacgcgggccaagtttgtgtttcaccaaccagatttatagtggaagaaagcgtgttcgaacgttttcgtgatggttttgcagagcgtgtcggtcggatcaaagttggaaatggtttggatcaggatgcgcagatgggaccgatggcaaatgcccgccgcccggaggcgatggatcgtctgatcggggacgccgtgactcgcggcgcaaggttgcatactgggggcgaacgtgtcggcaacgccggctatttttatgcccccacggttctgagtgaagtaccgctggacgcggctattatgaacgaagaaccgtttggcccggtagctctgattaatccattcggcggtgaggaagcgatgatcgccgaagcaaaccgtctgccgtatggcttggcagcctacgcatggacagatagcgcggcgcgggcaaaacgcttagcacgcgagattgagacggggatgctggggcttaattctaccatgattggcggcgcggattcgccattcggtggggtgaaatggtccggacacggttcagaggacggtcccgaaggtgttatggcctgccttgtcaccaaagcggtacatgaagggtaa |
| LigW gBlock | acacaagacctgaagaccggcggggagcagggttacctgcgtatcgccaccgaagaagctttcgccacgcgagaaatcattgatgtctacctgcgcatgatacgcgatggaactgctgataaaggtatggtatcattgtggggcttttatgcccagtccccttcagagcgcgccacccagatcttagaacgtctgttagatcttggcgagcggcgtattgcagatatggatgcgacaggcattgacaaggctattctagcgctgacctcgccgggcgtacagccgctgcatgacttagatgaagcacggacgctcgcaacccgtgcaaatgatactcttgccgatgcgtgccaaaagtatccagaccgatttattggaatgggcaccgtggccccgcaggatccggaatggagtgcgcgcgaaattcatcgtggtgcaagggaactgggttttaagggcatccagatcaacagccacacgcaagggcgctacttggatgaggaattctttgatccgatattccgtgccctcgttgaagtcgaccagccgctgtatattcatcctgccacttcgccagattccatgatcgatccgatgttggaagcgggcctggacggtgcaatcttcggcttcggtgtggagacgggcatgcatctgctgcgcctgatcacgattgggattttcgacaaatatcccagcttgcaaattatggttgggcacatgggcgaggcgctgccctactggctctatagactggattatatgcaccaggctggtgtgcgctctcagcgctatgaacgtatgaaaccactgaaaaaaaccatcgaaggttatcttaaaagcaacgtgttagtgacaaattctggagtcgcgtgggaacctgcgattaaattttgtcagcaagtaatgggtgaggatcgggttatgtacgcgatggactacccgtatcagtacgttgcagacgaagtgcgtgcgatggatgccatggacatgagtgcgcaaacgaaaaaaaaattttttcagaccaacgctgaaaaatggtttaaactttaa |
| LsdD gBlock | atggctcaatttccgaataccccaagcttcacgggattcaacacgccgtctcggattgaggcggatattgcagatctggcccacgaaggtacgattccgcaagggttaaacggcgcattttatcgtgtccagcccgatccgcagtttcctccacgcctcgatgatgacattgcctttaacggagacgggatgattacccgattccatatacatgatggccaggtcgacttccgtcaacgttgggcgaaaaccgataaatggaaactggaaaacgcggccggaaaagccctgtttggtgcctaccgcaacccactgaccgatgacgaggcggttaaaggcgagatccgttcgaccgccaacactaacgccttcgttttcggtggcaaactgtgggcgatgaaagaggacagtccagcactcgtaatggatccggcgacgatggaaaccttcgggttcgaaaagttcggcggtaaaatgacaggccagacctttactgcccatccgaaggtagatccgaaaaccggcaatatggtagcgatcggttatgctgcaagcgggttgtgcacagatgatgtgacctacatggaagttagtccggagggtgaattagtacgcgaagtgtggttcaaagtgccgtattattgcatgatgcacgacttcggcattacagaggattacctcgtgctgcacattgttccttccatcggaagctgggaaagattagaacagggcaaaccgcactttggctttgatactactatgccggttcacctaggtatcattccgaggcgtgacggtgtgcgccaggaagatatccgttggttcacgcgggataattgttttgccagtcatgtactgaatgcttggcaagaagggaccaaaattcactttgtgacttgcgaagcgaaaaacaacatgtttcctttctttccagatgtccatggcgcgccctttaacggtatggaggcaatgtcacatcctacggactgggtggtcgacatggcaagcaacggcgaggactttgctgggatcgtgaagctttccgatacagctgcagaatttcctcgcatcgacgaccggtttaccggccagaaaacccgccatggttggttcttagaaatggatatgaaacgaccagtggaattgcgcggtggttcagcgggcggcctgctgatgaattgtctgtttcacaaggacttcgaaacgggtcgtgaacagcattggtggtgcggcccggtttcgtctcttcaggagccgtgttttgttccgcgcgcgaaagatgcccccgaaggtgatggatggattgtgcaagtttgtaatcgtctggaagaacagcgttccgatttgctgatatttgatgcgctggatattgagaaaggcccggtggctacggtcaatatccccatccgcctgcgctttggcttgcatggtaattgggcgaatgcagacgaaattgggcttgcggaaaaggtcctggccgcagcgatcgcaggaagcgaaaatctgtattttcagagcgcattggcacatcaccatcatcaccatcaccattaa |
| FdhA gBlock | ctaagcgacaggcacgtcaaagggagaccgcatgaaatgaaaacacgcgccgcagttgcgtttgcgccaaagcaaccgttggaaattgtagaactggatctggaaggtcccaaagctggggaagttctggttgagattatggcgactggagtgtgtcacaccgatgcatatacgttagacgggttcgacagcgaaggcattttccctagcgtgctgggtcatgaaggtgccggtatcgtgcgcgaagtgggccctggggtaacttccgtgaaacctggcgatcatgtgatcccgctctatacgccggaatgtcgccagtgcaaatcgtgcttgtcgggtaagaccaacctgtgcaccgctattcgcgccacgcaagggcagggcctgatgcccgatggcaccagtcgtttttcttacaaaggccagaccgtgttccactacatgggttgcagtacattctctaattttacagttctgccagagatcgcggttgcaaagattcgcgaggatgcgccgtttaaaacctcatgttatattggctgtggcgtgacgacgggtgttggcgcggtgattaacactgctaaagtacaggtcggtgacaacgtcgtggtctttggattaggcggcataggtctcaatgttattcagggagcgcggcttgccggtgcagggaaaatcattggcgtcgatatcaatccagatcgggaggaatggggccgtaaatttggcatgactgactttctgaatagtaagggcatgagccgcgaggacgtagttgctaaagtcgtcgccatgaccgatggcggtgcggactatacctttgatgccaccggtaataccgaagtgatgcgtacggcgcttgaagcatgccatcgtggttggggaacctccataatcattggtgtggcagaggcgggtaaagaaattagcacgcgtccgttccaattagttactggccgtaactggcgaggcacggccttcggaggcgccaaggggcgcacagatgttccgaaaattgtagatatgtacatgaccggaaaaatcgaaatcgatccgatgatcacccatgtcatggggctggaagagatcaacacagcatttgatctgatgcacgctggtaaatcgattcgttcagtagtggtgttctaa |
| LigV gBlock | cagtttgaacgtatcaatccgatgacaggggcagtagcctcgcaggcagaggccatgaaagcgtcggacattccttccattgctgcccgcgcaggacaggcctttccggcgtgggcagcgatgggccccaacgcacgtcgcggcgtactgatgaaggcggctgcggcgttggaagcgcgggctgatgctttcgtcgaagccatgatgggcgaaatcggcgcgactagagggtgggcgctgtttaaccttggccttgcagcaagcatggtgcgcgaagccgccgcgctgaccactcaaatctctggagaggttattccatctgacaaaccggggtgtatttcgatggctctgcgcgaaccggttggtgtgattttgggcatcgcgccgtggaatgcgccgattatccttggggtgcgcgcaattgccgtgccgcttgcctgcggtaacgcggtgatattaaaagcaagcgaaacatgtccgcgaacccacgcgctcatcatcgaggcctttgctgaagcaggtttcccagaaggcgtggttaatgtagtgacgaacgcgcctgcagatgcagcggaagtggtcggggcgctgattgatgcgccggaagtgcgtcgtataaactttaccggtagtactaatgtaggcaggattatcgcaaaacgggcggccgagcatttgaaaccctgtttactcgaactgggcggtaaagcaccgttaatagttctggatgatgcggatctagacgaagcggtcaaagctgcggcttttggcgccttcatgaaccaagggcagatttgcatgtcaacggagcggatcatcgttgtagatgccgttgccgatgcattcgcagataaattcaaggccaaggtcgcctccatggctgtaggcgacccgcgtgagggtacgaccccgttgggtgcagttgtcgacgctaaaactgtcgctcattgccgtagcttaattgacgatgccctggcaaaaggtgcccgtctgctgaccggcggtgaaaccacgcacaatgtgctcatgcccgcccatgtcgtagatggcgtgacgcaggatatgaagctgttccgcgatgagagctttggcccagtggtgggcgtgattcgcgcgcgcgacgaagctcatgccattgaactggcgaacgacagtgaatatggactgtcagcggctgttttcacacgtgacacagcgcgcggcctgcgagttgcccgccagatccgtagcggtatttgccatgttaatggacctaccgtccacgatgaggcgcagatgccttttggtggagtgggtgcgtccggctacggtcgttttgggggtaaagccggcatcgatagttttaccgagctgagatggattacgatggaaacccaaccaggtcactatccaatttaa |
| Saro_0995 gBlock | aaagccgccgtactcgtcgaaccgggtaaaccgctggatattcagcatttaagcgtgagtaaacccggccctcatgaagtccttatacgcacagcagcctgcgggctgtgccatagtgacttgcacttcatcgaaggtgcctatccacatccgctgccggctgtgccagggcacgaggctgctgggattgtggaagcggtaggttcagaagtgcgcacagtaaaagtgggtgacgctgttgttacctgcctgtccgcgttctgtggtcattgcgagttttgcgtgaccggccggatgtcgctgtgtcttggtggcgatactcggcgcggtgcgggtgaggcacctcgcttgacacgcaccgacgatggaagcgcagtgaaccagatgctcaacctatcggcctttgcagaacaaatgctggttcacgaacatgcctgtgttgcgatcaatcccgagatgccgctcgatagagctgcggttatcggctgtgcggtaaccactggcgcgggtgcggtgtttaatgctgcgaaactgaccccaggagagacggtatgcgttgtcggctgtggcggcgtaggcttagcaacggtcaatgccgcgaaaattgccggggcaggccgtattatcgctgtggatccgatgccggaaaaacgcgaactggccatgaaactgggtgcgaccgatgtgatggacgcgggacccgatgctgcggcacagatcgttgaaatgacgaaaggcggcgttcaccatgcgatcgaggccgtggggcgtcctgcatctggcgaccttgcggtcgcgacgctgcgtcgtgggggcaccgccacgattttaggtatgatgccgctggcacacaaggtcggattatcagcgatggatctgctgagcgataagaagctgcagggtgcaattatgggccgcaaccacttcccagtggatctgccgcgactggtcgacttctacatgcgtggcttgttggatctagacactatcattgccgaaaggattccgcttgaagggataaacgatggttttgaaaaaatgaaacagggacattccgcccgttctgttattgtgtttgatcaataa |
| Saro_1431 gBlock | acaatcaatacaattcgcgtacgttcgccggccactctcgacaccttaaatttcgatacgctgacggattgtggacaaccgggaacgagcgaaatccgcattcgtctgcgcgcaacttctctgaacttccactactacgcgatgattaccagaatgctgccggctgcaacaagtcgaattcctatgtctaacggcgcctgacaggttttcggggtgtgcgatggcgtgaccaaattccaggcgcgtaacgcagttatctcgacctttttcaccgacaggaacgccggtccgccacagtcagccgcgtttacgaccgtcacggctgatgggattaatcgctacgcgcgggaagaagtggtggccccggctcattggtttacccgcgcgccgttatgctatagtcacgcaaaagccgccacgctgacctgcgcgggccttactgcatggcgtgctttgttcatagataacgctatcaagccgggcgacacggtcttggtgcagggcactggcagcgtttcggttttcgcgctgcagttaacaaaggcggcatgcgcgcgtgtcatcgcaacgagttcctcccaccagtaactgaaacgcctgcgcagccttagagcgaataaaaccataaactataaaacgcaaacctcacgggggatgcagacactagatttcactgccggtatttgtgtacactgtattgtcgagattagccggcccggtacgtttcatcaagcgatgatgtccacccgcgtgcgtgctcatatcgcgctgatcggtgttctcgcgcgttttgcgggtccagtttaaaccactttgctgatggcacagaatctgcgcgtataaggccttaccgtggcctcacgtaccaatcatctgcgaatgattcccggtatcgaggcaaaccgtatccaacctgtcattcaccgccattttccatttccgtattttgccgctgcctttcgccatcaacagagctgccgtcatttttgttaaatcgtgattgacatttga |
| Saro_1476 gBlock | ttgggacgtgcatcggtgctggtaaaaccgaaccaactggagacgtgggatgttaaagtagccgatccggaaccgggcggtgccttagtttcgattgtgctgggtggggtatgcgggagcgacgtccatatattgaccggcgaggctggcgtgatgccgtttccgatcattctgggacatgagggcgtgggaaggatcgaaaaactggggcacggcgtcagcactgattacgctggtgaggaacttaaacccggcgatctggtatattggtcgccgattgctctgtgtcatcgatgttattcctgcaatgttctcgatgaaacaccttgcgaaaatacccagtttttcgaagatgcttccaagccgaactggggttcatacgcagattatgcatggctgcccaacggtatgccgttctataaactgccagcccaagcgcagcctgaagcggttgctgcgcttggctgtgcacttccaaccgccctgcgcggctttgatcgctgcggcagtgttagagtgggtgaaactgtggttgtccaaggtgcaggccctgtcggcctgtctgcagtgctcgtggcggcgcaggccggggcgcgtgacgtgattgttattgacggttcaccacttcgtcgcgaagcggctaccgcattgggtgcctctctgacgattggcttagatgtcgcgcctgaggaacggcgccggatgatttacgatcgcgttggtcgcaatggtcccaatgtagtcatcgaggcagccggagttctgccagcgtttccggaaggggtggacctgaccggtaaccacggccgttacattgtgctaggattgtggggcgcaatagggacccagccgatcagcccgcgcgacttaacaatcaaaaacctgactatcgctggtgcgaccttccctaaaccaaaacattattatcaggccttgcatttagcgacggccctgcaggaccgtgtaccgttagccggtctggtgagccaccgttttggcgtcagccaggcgggcgaagcgctgagtctcaccaagagtgggacagcgattaaggccgtgatcgatccgacgatcacgtaa |
| Saro_2795 gBlock | gcggcaattaatcttccccgcgtgattcgtgctggtgggggtgcattagccgaactgcccgatgcaatggcgcagtgcggcctttcacgcccgttcgtggtgaccgatgcattcttagtgcaaagcgggatggtcgctcggatgttagaggttctggacggcgctgggattgcggccacggtcttcgatgctacggtacctgatccgactgttgctgtggtagaacaggcgcttggcgcattgcgagaggcggaatgtgattgtgtgatcgggtttggaggtggtagcccgatcgacaccagtaaagccattgccgccctggcgctggaaccgcgtgcagttcaatccatgaaggcaccagcgacgaccgacgtcccgggtctgccgatcattgccgtcccgacgaccgccggcaccggctcggaggcgactaaatttacaatcgtgaccgatgaggcgacgagtgaaaaaatgctctgcgcaggtctggccttcctgcctactatagccattgtagatttcgagctgaccatgggcaaaccggctcggctaactgccgacacaggtattgattcgctgacacatgcgattgaggcctatgtttctaagaaagccaatccgtttagtgatgctatggcgatctcggcgatgaaactgatcgcgccgaacattcgcaccgcctgcgccgaacccggaaaccgtgctgcacgcgaagcgatgatgattggcgcgcaccatgccggtattgcgttttccaacgctagcgttgcactggtgcacggtatgagccgcccaatcggcgcattctttcatgtgccgcacggattgtccaacgcaatgttgctgcctgcgattaccgcgttttccgctccgtcagcgttaccacgttacgccgattgtgcccgtgcgatgggtgtagctttggaaagcgaaggcgaccagtctgccgttgcaaggctgctcgacgaactggcggcgctgaacgcagaccttagtgtcccgacgccgcagtcgcatgggatcagcgctgatcgttggtttgaagtagtgcctgaaatggcgagacaggcaatagcatcaggctctccaggcaataatccacgcgttcctgatgcggcggaaatcgagcgcctctatgccgaagtctttggctaa |
| Saro_2870 gBlock | cgattgaaagttctgggacttatggcagcactgctgccgctggcggcttgtaacatcaaaagcgagggtggaggggatgcagtcgccaacgctggagtcacagatgccctgattgcccaagcgcccgaaggcgaatggctgagctatggccgcgattatggggaacaacgcttttcaccgttgacccaaattaatgatggtaacgtcgggcagttgggtcttgcctggtttcatgacctggagactgcgcgcgggcaagaagcgacgccgctgatgcatgatggtacgttatatatctcgactgcgtggtcaatggtgaaagcgttcgatgcaaaaaccggcgcgctgaaatggagttacgatcccgaagtaccgcgtgaaacgctggtgcgcgcatgctgcgacgcggtcaatcgtggcgtcgcgctgtatggagataaagtttttgtaggtacgctcgatggtcgtctagtagcgttagatcagaagaccggaaaagtagtttggtccaaggtagtagtgcccaatcaggaggactacaccataactggtgccccgcgcgtggtgaaaggcaaagttctgattggtagcggtggctcggagtacaaagctcgaggctatattgccgcctatgacgttaacacaggcaacgaagtgtggaaattccacaccgtccctggcaatccagcggatgggtttgagaacaaagcgatggaaaatgccgctcgcacttgggctggtgaatggtggaaactcggtgggggtggcacggtgtgggattccatcacctatgatccagccaccaacctagttctgttcggcacaggcaatgcagaaccatggaacccggcagcagccgggcgggagggagacagcttgtacacgtcctctattgtagcggtgaatgccgatactggcgactatgtatggcattttcaagaaaccccggaagaccgttgggacttcgattccgcgcagcagattacgctggccgacctgacaattgatgggcagcggcgccacgtgatccttcatgcgcctaagaacggtcatgtttatgtgttggacgcaagaaccgggcagtttctgtcggcaacgccctttgtgatggtgaactgggcgaccggtattgatcctaaaacgggcaaggccactgtcaatccagaagcccgttatgaaaaaaccggcaaacctttcgttagcctgccaggtgcggtaggcgcacattcatggcagccgcagagtttcagcccgaaaaccggcctgctgtaccttccggtgaacaatgcggcatttccttatgcagccgccaaagactggaaagcaaccgatattggtttccagaccggtctcgacggctatgttaccagtatgccagccgacgcaaaggtccagggcgcagcgatgaaagcgaccactggtacgttagtggcgtgggacccggttgcgaagaaagccgcttggaaagtcgaactgccgagcccgagtaacggtggcattttatcgacagctggcaatttagtgtttcaaggtaccgcgggcggtgattttgttgcatacaacgccgataagggcaaacaattatggtcttttccggcgcagagtggcatccttgccgcgccgatgacctatgctatcgatggggaacagtacgttgcggtcatggtgggctggggaggtgtgtgggacgtcgccacaggtgtgctcgctcataaggccaaaaaacagaggaacataagccgcctggtagtgttcaaactgggcgggaaagccacgctgccggctgctcctccgatggcaaaaatggttttggatccgccgccgtttacaggtacgcccgaacaagctaaggccggtggcgaattatacggacgttactgcaacgtttgtcatggtgatgctgcggttgcgggcggcgtgaatccagatctgcgtcactcagctgcgcttaatgcaccagaggcgatccggtctgtggtgattgagggggcgctgcagcacaacgggatggtctcgttcaaatctgcgctgaagcctgaggatgcggataatatccgccactacttgatcaaacgtgcaaatgaagacaaagctctcgaagccaaaggaggctaa |
| Saro_3463 gBlock | attccgcatggtgaacattcaatgctggcaatgcagttggatggtccaggcaaacggctgcacccagtcgtgcgccctctgccgttaccggggcgaggtgaagtgcgggtaaaagtgcatgcctgtggtgtttgccgtacggacctgcacgttgcagatggcgatattcacggtctgctacctattgtgccggggcacgaagtgataggcgttgtcgatgcactggggccgggggtgacggatgttgaacctggtgcgcgtgtaggtgtcccgtggctcggccatgcctgtggcacctgcccatattgcgacagcgggagggaaaacctttgtgatgcgccgctgttcaccggttttactcgcgatggcggatacgctacccatgtgattgcagatgcgcgcttttgctttcctattccagagggttttgacgatctgcacgcggcgccgctcctgtgcgcgggcttgatcggctatcgcgctcttcggcttgccggcgatgcacctgtactcggattctatggttttggagcggcggcgcatattttagctcaggtggccctgtggcagggtagaacggtttacgcgtttactcgcgatggcgacgctaaggcccaggcctttgctcgtgacatcggttgccaatgggccggaccctctggcgctgcgccgccgcaagctctggacgcagcgatcatcttcgcctccgcgggagaattggtgccgacagccctgcgtgcagtgcgcaaaggcgggcgtgttgtctgtgccggtattcatatgagcgatatcccggcattcccctacgccgatttatgggaggaacgtcagatcctgtcggtagcgaatttaacccgacgcgatggcgtagaattcctgccccttgcagcgcgtgcaggcgttcgcacacatgtcgaggccatgccgttaatgaaagcgaacgaggccctggaccgcctgcgtcgtggcgacgtcagtggcgctctggttttggtgccataa |
| Saro_3899 gBlock | gacgcatacgctgcaattatcgagcgtcagggtggagaattcgttctggataacgtatctatcgaggatccgcgcgatggcgaagtgctggttaaggttgccgcagctggcatgtgtcataccgatctgacggttcgcgatcaatattacccgacgccgcttccggcggtgctgggccacgaaggtagcggcgttgttgaaaaagtgggacgtggcgtcaccactgtcaaaccaggtgacaaagtagtgttatccttcagctattgcggtacttgtccttcgtgcctcaaagggcatcaggcatactgtccgagcctgttcccgttaaatttcatgggccgtcgcctggatggttcaacgcccattacacgcaacggtcaagaggtcaacgcctgctttttcgggcaatcctcttttgcgacctatagtattgcgtcagaaaacaattgcgtcaaggttgccgacgatgcacagattgaacttttgggcccactgggctgcggcattcagaccggtgcgggaagtattttaaatgctctttgtcccgaacctggttcctctatagcgatctttggggtggggagtgtaggcttaagcgccgtgatggctgctaaagcatcgggctgcttgaagatcatcgcggttgacagaaatgcaggtcgcttggaactggcgcgtgaactgggcgccaccgatgtgattgacgccaacacggtcaatgctcaggaagcgatcgtcgcgatgactggtggcggcgccgactatgcaatggataccacagccattccagcggtgctgcggagtgcggtggatagcacgcacaatatgggtgaaacagcagtggtgggcggggcgaaactgggtaccgagttttcactagacatgaataacatgctgtttggtcgaaaattgcgtggcgtagtcgaaggatcgagcacgcctcaggtgttcatcccgcaactgattgcgatgcagaaagccgggctgtttccgtttgagaaactctgtaccttttatgatctggatcagatcaaccaggccgtagaggataccgaaaagactggaaaagcgataaaagccattctcaaaatgtaa |
| Saro_0060 gBlock | tctacacagcctgcaaccatagctgattccgcgaccgatctggttgagggtcttgcacgtgcagcccgttctgcgcagcgccagttggcgcggatggattcaccggtaaaagaacgcgcgctgacgttagccgctgcagcgctgcgtgccgctgaggccgaaattttagccgctaacgcgcaggatatggcgaatggcgcagcaaacggcctgtcctcggccatgctcgaccggctgaagttaacgccagagcgtctggccggcattgccgatgctgtggcgcaagtcgccgggctggccgatccggtcggcgaggtgatcagtgaagctgcgcgtccgaatggcatggtgctgcagagagtgcgtattccggtcggagttatcggcatcatttacgaaagccgccccaacgttaccgccgatgcagcagcgctctgcgtgcgttcaggtaatgcggcgattctgcgcggtggctcggaagcggttcatagtaaccgtgcgatccataaagcgctggttgctgggcttgccgaaggcggagtgccggcagaagcggtgcagcttgtacctacgcaggaccgtgctgccgtaggggcaatgctaggtgccgcgggactgatcgacatgatcgttccgcgcggcggaaaaagccttgtcgctcgcgtccaggcagatgcccgcgtgccggtgttagcacacttggacggtatcaaccacacgtttgttcatgccagtgcagatccggcgatggcccaagcgatagtgttgaatgccaaaatgcgtcgcaccggcgtttgtggtgcgatggaaaccctgctgattgacgcgacttatccagatccccacggcctggtcgaaccgctgctagacgccggttgcgagctgcgcggcgatgctcgagcgagagcaattgatccgaggattgcgccagctgccgacaacgactgggatacagaatatttggaagcgattctttcggttgcagtggtcgacggtttggatgaagcgctcgcccacatcgcgcgccatgcctctggtcataccgatgcaatcgtcgcggcggaccaagatgtggcagaccgattcttagctgaagtagatagcgcaattgtaatgcataatgcatccagccagtttgctgatggcggtgagttcggcctgggtgctgagattggtattgccacggggcggctgcacgcgcgcggccctgtagcgctcgaagggctgactacctacaaatggctggtgcgcggaagcgggcaaactcgtccataa |
| Saro_1104 gBlock | cgcgaacggctacagcaatacattgatggaaagtgggtagacagtgaaggtggcaaacgtcacgaagtcattaatccgactacagaggaaccctgttgtgtgattacgctgggcacgcaagcagatgtcgacaaagcagtggccgcggcacagcgcgcctttaaaaccttcagcaaaacgacgcgtgaggaacgactggcgctgcttgaacgcatcgtagaagaatacaagaagcgtgtccctgatttagccgccgcgatggccgaggaaatgggagctccggtaagctttgccagcaccgcgcaagttggcgccggaatcggagcatttctgggcaccatggccgcgctccgtaatttctcctttgttgaggacaacggtgcgtttaaagtggcctacgaaccgataggtgttgtgggtatgattacgccatggaactggccactgaatcagatagctctgaaagtagcaccggcgctggccgcggggaataccatgatcctgaaaccgtccgaggaatgcccaaccaacgcagcgatctttaccgaaattttggatgccgcaggggttccgccaggggtttttaacctgattcagggcgatggtcctggtgtaggcactgcgatcagtagtcatccgggcattgatatggttagtttcaccggttcgacccgtgcgggcatcctcgtggcgaaagctgcggccgataccgtcaagcgggtgcatcaggaacttggcggtaaatctcccaatgtggtgctgcccgatgcagacttcgcaaaatatctgccgtctaccgcgtcaggcccgttggtgaacagcggccagagctgcatttcgccaacccgtattttagtaccaagagaacgcgaagcagaagccgcggcttttgtttctgcgatgtactccgcaacaccggtcggggatccgatgcaagaaggtgcgcacattgggccggtggttaacaaagctcagtttgacaagatccgcggtctgattcaatcggcaatagacgaaggcgcgaaactcgagacaggcgggcccgacttaccggccaatgtgaaccgcggctattatatcaaaccaacggtcttttcaggcgttactcctgatatgcgcattgctcaggaagaaatcttcggcccggtggcgacgattatggcgtacgattcattagaggaggccattgagatcgcaaatgatacagcctatggactgtcggcctgcattactggtgatccggcgaaagcggctgaagtcgctcctgagcttcgtgcaggtatggtggctatcaataactggggccctactccgggtgctccgttcggtggctataaacagtccggtaacggtagggagggagggttgtatgggttgaaagacttcatggaaatgaaagcgatcagcggcctgcctgcctaa |
| Saro_1197 gBlock | actgcccctaccgccgcagacctttccgccgatattgcacgggtttttgcactgcaacaagcgcacatgtgggaggccaaggcgtccaccgcggcggagcgcaaagaaaaattggcgcgtctgaaggccgcggttgaagcacacgcggatgacattgtggccgcggttctggaagatacgcgcaaacctgttggtgaaataagggtgaccgaagttctgaatgtaaccgccaatatccagcgaaacatcgataatctcgatgaatggatgaaaccggtcgaggtcgctacctcactgaatccagcggaccgcgcgcagataattcatgaagcgcgcggcgtatgcctgattcttggcccatggaatttccccttaggtctggcgctgggtccggtcgccgctgctatcgccgcaggcaatacttgtatcgtgaaattaacggacttgtgtccagcgaccgcaagagtggcatcggtgatcgtgcgtgaagcgttcgatgaaaaagatgtggctctgtttgagggagacgttagtgtagctaccgcgcttttggatctgccgtttaatcatgtattttttacaggctctccacgtgtaggcaaaattgtgatggctgctgcggcaaagcatctgaccagcgtcacgttagagcttggtgggaagtctcccgttattgtcgatgatagcgcagatatcgatcaagttgctgcccagttagccgcggccaaacaattcaacggcgggcaggcctgcatttccccggactatgtgtttgtgaaagaagacaaaaaagctgcgctggtagaaggtttccgtgccaatgtgcagaaaaacttgtatgatgatgcaggcaacctgaaaaaagacagtattgcacaggtggtcaacaaagcgaactttgatcgtgtgaaagccatgttcgacgatgcagtcgcaaaaggcgcgaccgtcgccgctggtggaacgtttgaagcggatgacttgactattcatccgacaatgctgacaggcgtaaccccgcagatgactattctccaggatgagatctttgcccctgtcattccggtgatgacctacgacacgctggatcaagcgatcgggtatatcgaagcacgcgacaaaccgctagcactctatgtttacagtaaagatgaagcgaacgttgaaaaggtcttagcccgcacgtcatcgggtggtgttacggtgaatggtgtgttctcgcactacctggaaaacaacctgccgttcgggggggttaacacaagcggtatgggcagctaccatggcgtgttcggatttaagtgctttagccacgagcgggctgtatatcgtcatcagcagtaa |
| Saro_1410 gBlock | ggttaccgggttgtagtggtgggtgcgactgggaatgtggggcgtgaaatgctgaacattctggcagaacgcgagtttccttgtgacgagatcgcagcggttgctagctctcgttcgcagggcaccgaaatagaatttggcgaaactggccggaagctgaaagtacagaatgttgaaaattttgattttaccggatgggacattgcactgtttgcggcgggatcaggcccgacgcagatccatgctccacgtgccgcttctcagggctgcgtggtgatcgataacagtagcttataccgcatggacccggacgtgcctctgatcgtgcccgaggtgaatccggatgcgattgatggctataccaaaaaaaacattattgccaatccaaactgttccaccgcgcaaatggtcgtggcgctgaaaccgttacatgatgccgccaaaattaaaagagttgtcgtctccacgtatcaaagcgtttccggcgcgggtaaagaagggatggatgaactgttcgaacaaagccgcgcgatatttgtcggggacccggtggaaccgaaaaaattcaccaaacagatcgcattcaacgtgatccctcatatcgatgtattcctagacgatggttcgactaaagaagagtggaaaatggtcgccgaaaccaaaaaaattttggaccccaaggttaaggtaacggcaacctgcgtgcgtgtgccggtgttcatcggccactcggaagcgttaaacattgagttcgagaatgaaattagtgccgaggaagcgcagaatatcctgcgcgaagcaccaggtgtgatgctcgtcgataagcgcgagaacggcggatatgttacgccggtcgaatgcgttggtgattttgccacatttgttagccgcgtacgtgaggattcaacagttgataacggccttaatatttggtgtgtcagtgataacctgaggaaaggtgctgccttgaacgctgtacagattgcagaactgctcggtcgtcgacaccttaaaaagggttaa |
| Saro_1967 gBlock | gcgatcaaagttgcgataaacggttttggacgtatcgggaggaatgtggcccgcgccattttagaacgtcccgattgtgggttagaactggttagcattaacgacctggctgatgccaaggctaacgccctgctgtttaaacgcgacagcgttcatggcgcgttcagtggcgaagtatcagtggatggcaatgatctgattgtgaatggcaagcgcattcaggtgactgcagagcgcgatcctgctaacctgccacacggagccaatggtattgacattgcgctggaatgcacgggctttttcaccaatcgtgatggtggccagaaacacttggacgcgggcgccaaacgcgttctgatttccgctccggcaaaaaacgtagacctgacggtcgtctatggtgtgaaccacgacaaactgaccggcgatcataagatcgtgtccaacgcgagttgcacgaccaactgtttggcgccgatggcaaaagtcctgcatgaatctatcgggattgagcgtggtctaatgacaacgattcattcgtataccaatgatcaaaaaatactcgaccagatccatagcgatcctagacgggctcgggcagcggcgatgaatatgatccccacaagcaccggggccgcagttgcagtgggtgaagttctgccagacttaaaagggaaacttgatggttcgtcgattcgagtcccgaccccgaacgtatctgtcgtggatcttactttcacgccgaagcgtgataccagcgtagaggaagtaaatggtctcttgaaagcggctgccgaaggcgcattgaaaggcgtgttaggttacaccgacgaaccgctggtttcaatcgattttaaccacgatccgcatagttcaacaatcgacagccttgagactgccgtgctcgaaggtaaactggtgcgcgtcctgtcttggtacgataatgagtggggcttttccaaccgtatgctggatacggcgggagcaatggcgaaattcctttaa |
| Saro_2869 gBlock | aatgacatgactaccatctcacgcacgcagcgtgaatactccgaggccgcaaaagctttcctcgcgagaaagccgcaattgtttattaataacgagtgggtcgatagcagtcacgatgcagtgatcgaagtggaagacccctcgaatgggaggattgtaggtcatgtcgttgatgcctcggacaaagacgttgaccgggcggttgccgctgcgcgggccgctttcgatgatggtcgttggtccaacctgccgccaatggtacgcgatcgtaccatgaatcgcctggccgacctgcttgaagcaaacgcagatctctttgcagagctggaagcgattgataatggtaaaccgaagggtatggccggcgccgttgatattccaggtgcgataagccaactacgcttcatggcaggatgggccagcaaggtagctggcgaaacgacgcagccttacacgatgccgaatggcaccgtgtttagttacaccgtcaaagaacccgtcggtgtctgcgcgcagattgtgccgtggaacttcccgctgctgatggcatcattgaagatcgccccggcgctggcggctggatgtacactggtgctgaaacctgccgaacagacatcgcttaccgcgttaaaactggcagatttggtggttgaggctggctttcctgcgggagtgatcaacattatcacagggaacggccacaccgcaggtgatcgcatggtcaaacatcccgacgtagacaaagtcgcctttactggctccaccgaaatcgggaaactgataaatcgaaacgcaaccaccacgcttaaacgggttacgctcgaactgggcgggaaaagtcccgtagtggttatgccagacgtagatgtggcgcagaccgcgcctggcgttgccggtgcgatttttttcaacgctggccaggtttgtgttgccggtagtcgtttatatgcgcaccgttcggtgttcgattccgtgttagaaggtatgacccagactgcgccgttttgggcgccgcgcccgagcctggatccagaagcacacatgggaccgttggtcagcaaagagcaacatgaccgtgtgatgggatatatcgaggcgggcaagcgtgatggcgccagcgtagtgatgggcggtgattgcccaagcgctgatggagggtactatgttaatccgacgattctggcagacgtgaatccgcagatgtctgtcgtgcgcgaggaaatttttggtccggttgtcgtcgcccaacgcttcgacgatttagatgaagtggcgaaaatggcaaacgacacctgttttggcttaggtgcgggcgtgtggacgcgcgatgttgcggtgatgcataaacttgcttcaaagatcaaatctggcactgtgtggggcaactgccatgccctgatcgatacagcgctgccttttggcggctataaagaatctgggctgggtcgagaacaggggcgtgccggtattgatgcttatttggagactaaaacagtaattattcaaatgtaa |
| Saro_3848 gBlock | gctacgcagttgagaagtgcagaaaatgaatatgggatcaaatccgagtatggtcattatataggaggtgagtggattgcaggggatagcggcaagaccatagatttactaaatccctctaccggtaaagtgctgaccaaaattcaagccggcaacgcaaaagatattgaacgcgcgattgccgctgcaaaagcggcgtttccgaagtggagccagagcctgccaggggagcgccaagaaatcctgatagaggttgcgcgtcgtctgaaagcacgccattcgcactatgcaaccttagaaacgctcaataacggtaaaccgatgcgcgaatcaatgtatttcgatatgcctcaaacgatcgggcaatttgagctgttcgccggtgccgcctatggcctgcatggccagacgctggattatccagacgcgattggcatcgtccaccgtgaaccgttaggcgtatgcgcgcagattattccatggaacgtgccgatgttgatgatggcgtgcaaaatcgcgcccgcgctggcctctggcaacactgtcgttctgaaaccggccgaaacggtgtgcctttctgtgattgaatttttcgtggaaatggctgatctgttgcctccgggtgtgatcaacgttgttaccgggtatggtgctgacgttggcgaggcgcttgtaacaagccctgatgtagctaaagtggcctttaccggttcgattgctacggcgcgccggattattcagtatgcctcggccaatatcattccacagacgctcgagttgggcggtaaatcagcgcatatcgtgtgtggcgatgccgatattgacgcggcggtggaaagtgcgactatgtccaccgttttaaataaaggtgaagtctgtctggctggttcacgcctgtttctgcatcagtccatccaggatgaattcctggccaaatttaaaacagcgcttgaaggcattcgccaaggcgacccgctagatatggcgactcaacttggagcgcaggcatcgaagatgcagtttgacaaggtgcaaagctacttaaggctggctacagaggaaggggcagaggtactgaccggcggtagtcgttcagatgccgcagatctggcagatggcaattttatcaaaccgacggtttttactaacgtcaataactccatgcggatcgcgcaggaagagattttcggaccggttaccagcgtaattacatggagcgacgaagacgacatgatgaaacaggccaacaatacaacttacggcttggctggcggtgtctggaccaaggacatcgcacgagcacaccgtattgcgcgtaaactcgaaactggcacggtctggatcaatcgctactacaacctgaaagccaacatgccgctgggaggttacaagcaaagtggctttgggcgtgaattcagccatgaagtgctgaatcactacacccagaccaaatctgtggttgtcaacctccaggaaggtcgtaccggaatgttcgatcagtaa |

**Supplementary References**

1. Cecil JH, Garcia DC, Giannone RJ, Michener JK. 2018. Rapid, Parallel Identification of Catabolism Pathways of Lignin-Derived Aromatic Compounds in Novosphingobium aromaticivorans. Appl Environ Microbiol 84.

2. Perez JM, Kontur WS, Alherech M, Coplien J, Karlen SD, Stahl SS, Donohue TJ, Noguera DR. 2019. Funneling aromatic products of chemically depolymerized lignin into 2-pyrone-4-6-dicarboxylic acid with. Green Chemistry 21:1340-1350.

3. Gonzalez CF, Proudfoot M, Brown G, Korniyenko Y, Mori H, Savchenko AV, Yakunin AF. 2006. Molecular basis of formaldehyde detoxification. Characterization of two S-formylglutathione hydrolases from Escherichia coli, FrmB and YeiG. J Biol Chem 281:14514-22.

4. Leonhartsberger S, Korsa I, Bock A. 2002. The molecular biology of formate metabolism in enterobacteria. J Mol Microbiol Biotechnol 4:269-76.

5. Kontur WS, Bingman CA, Olmsted CN, Wassarman DR, Ulbrich A, Gall DL, Smith RW, Yusko LM, Fox BG, Noguera DR, Coon JJ, Donohue TJ. 2018. Novosphingobium aromaticivorans uses a Nu-class glutathione S-transferase as a glutathione lyase in breaking the beta-aryl ether bond of lignin. J Biol Chem 293:4955-4968.

6. Vilbert AC, Kontur WS, Gille D, Noguera DR, Donohue TJ. 2024. Engineering Novosphingobium aromaticivorans to produce cis,cis-muconic acid from biomass aromatics. Appl Environ Microbiol 90:e0166023.

7. Blodgett JA, Thomas PM, Li G, Velasquez JE, van der Donk WA, Kelleher NL, Metcalf WW. 2007. Unusual transformations in the biosynthesis of the antibiotic phosphinothricin tripeptide. Nat Chem Biol 3:480-5.

8. Doherty AJ, Ashford SR, Brannigan JA, Wigley DB. 1995. A superior host strain for the over-expression of cloned genes using the T7 promoter based vectors. Nucleic Acids Res 23:2074-5.

9. Schäfer A, Tauch A, Jager W, Kalinowski J, Thierbach G, Puhler A. 1994. Small mobilizable multi-purpose cloning vectors derived from the Escherichia coli plasmids pK18 and pK19: selection of defined deletions in the chromosome of Corynebacterium glutamicum. Gene 145:69-73.

10. Gall DL, Ralph J, Donohue TJ, Noguera DR. 2014. A group of sequence-related sphingomonad enzymes catalyzes cleavage of beta-aryl ether linkages in lignin beta-guaiacyl and beta-syringyl ether dimers. Environ Sci Technol 48:12454-63.
